# Myoglobin and C-reactive protein are efficient and reliable early predictors of COVID-19 associated mortality

**DOI:** 10.1038/s41598-021-85426-9

**Published:** 2021-03-16

**Authors:** Ashaq Ali, Muhammad Noman, Yong Guo, Xu Liu, Rong Zhang, Juan Zhou, Yang Zheng, Xian-En Zhang, Yong Qi, Xiaohua Chen, Dong Men

**Affiliations:** 1 State Key Laboratory of Virology, Wuhan Institute of Virology, Center for Biosafety Mega-Science, Chinese Academy of Sciences, Wuhan, 430071 China; 2 Department of Laboratory Medicine, General Hospital of Central Theatre Command, PLA, Wuhan, 430070 China; 3 Medical Department, General Hospital of Central Theatre Command, PLA, Wuhan, 430070 China; 4 College of Life Science, Engineering Research Center of the Chinese Ministry of Education for Bioreactor and Pharmaceutical Development, Jilin Agricultural University, Changchun, 130118 China; 5 Department of Laboratory Medicine, General Hospital of Southern Theatre Command, PLA, Guangzhou, 510010 China; 6 Department of Biochemistry and Molecular Biology, Medical College, Hubei Minzu University, Enshi, 445000 Hubei China; 7 National Laboratory of Biomacromolecules, CAS Center for Excellence in Biomacromolecules, Institute of Biophysics, Chinese Academy of Sciences, Beijing, 100101 China; 8 Joint Expert Group for COVID-19, Wuhan Huoshenshan Hospital, Wuhan, 430100 Hubei China; 9 University of Chinese Academy of Sciences, Beijing, 100049 China; 10 Department of Pathology, General Hospital of Central Theatre Command, PLA., Wuhan, 430070 China

**Keywords:** Biomarkers, Health care, Medical research, Risk factors, Signs and symptoms

## Abstract

Since the emergence of SARS-CoV-2, numerous studies have been attempting to determine biomarkers, which could rapidly and efficiently predict COVID-19 severity, however there is lack of consensus on a specific one. This retrospective cohort study is a comprehensive analysis of the initial symptoms, comorbidities and laboratory evaluation of patients, diagnosed with COVID-19 in Huoshenshan Hospital, Wuhan, from 4th February to 12th March, 2020. Based on the data collected from 63 severely ill patients from the onset of symptoms till the full recovery or demise, we found not only age (average 70) but also blood indicators as significant risk factors associated with multiple organ failure. The blood indices of all patients showed hepatic, renal, cardiac and hematopoietic dysfunction with imbalanced coagulatory biomarkers. We noticed that the levels of LDH (85%, P < .001), HBDH (76%, P < .001) and CRP (65%, P < .001) were significantly elevated in deceased patients, indicating hepatic impairment. Similarly, increased CK (15%, P = .002), Cre (37%, P = 0.102) and CysC (74%, P = 0.384) indicated renal damage. Cardiac injury was obvious from the significantly elevated level of Myoglobin (52%, P < .01), Troponin-I (65%, P = 0.273) and BNP (50%, P = .787). SARS-CoV-2 disturbs the hemolymphatic system as WBC# (73%, P = .002) and NEUT# (78%, P < .001) were significantly elevated in deceased patients. Likewise, the level of D-dimer (80%, P < .171), PT (87%, P = .031) and TT (57%, P = .053) was elevated, indicating coagulatory imbalances. We identified myoglobin and CRP as specific risk factors related to mortality and highly correlated to organ failure in COVID-19 disease.

## Introduction

The COVID-19 pandemic has already become a challenge for the whole world which has dramatically paralyzed the socio-economic life^1^. The early categorization of COVID-19 patients is mainly based on the identification of effective laboratory biomarkers, which can efficiently predict disease severity^2^. To be able to guarantee timely treatment, it is crucial to define appropriate laboratory biomarkers capable of categorizing patients based on their risk. The key mechanism for SARS-CoV-2 invasion is the binding of the virus to the angiotensin-converting enzyme 2 (ACE2) membrane-bound receptor and the host cell's internalization of the complex^3^. ACE2, a glycoprotein and metalloprotease, is present both in membrane-bound as well as in soluble forms^4^. The membrane-bound form is comprised of a transmembrane domain which anchors its extracellular domain to the plasma membrane, whereas; it is degraded and secreted in its soluble form, while the circulation of the N-terminal ectodomain is scarcely detectable^5^.

Currently, personalized medicine demands model-based predictions for each person, where, the diagnosis of a clinical outcome is derived from observation (clinical characteristics) based on a large population size^6^. Zhou et al. has quoted an interesting summary of such prediction models, which spans the development of a clinical problem, statistical design and collecting data, followed by spotlighting, building, validating and finally evaluating the efficiency of those prediction models^7^. Statistical regression models are usually aimed at assessing the effect of clinical parameters, inferring the relation among the predictors thereby getting an insight into the population. Nonetheless, these models can efficiently predict the prognosis of a disease in its early stages which can be compared for the accuracy^8,9^. Furthermore, statistical models are based on a probabilistic and inferential theory holding well-defined properties which along with large dataset could also fit a small one. For prediction purposes, these two different strategies complement each other and should be used by integrating both of them^10^.

The analysis of recently published studies reveals the role of systemic vasculitis and cytokine-mediated coagulation disorders, predominantly responsible for multi-organ failure in patients with severe COVID-19 complications. The hematological (lymphocyte count^11,12^, neutrophil count^13^, neutrophil–lymphocyte ratio (NLR))^14,15^, inflammatory (C-reactive protein (CRP))^16^, immunological (interleukin (IL)-6)^17^ and biochemical (D-dimer^18^, troponin, creatine kinase (CK)^19^ biomarkers, as well as procalcitonin (PCT)^16,20^, erythrocyte sedimentation rate (ESR)^21^, aspartate aminotransferase (AST))^22^, and those particularly related to coagulation cascades in disseminated intravascular coagulation (DIC)^23^ and acute respiratory distress syndrome (ARDS)^24^ have been reported to be important biomarkers associated with COVID-19 disease. New laboratory biomarkers could be identified through the accurate analysis of multicentric case series; in particular, homocysteine and angiotensin II could play a significant role in this regard^24^. Some studies have reported elevated WBC, CRP, LDH, CK, and troponin associated with the severity of COVID-19^25,26^. Dry cough, fever and fatigue commonly occur in the early stages of COVID-19, however, ARDS, acute respiratory failure, multi-organ failure, and sepsis are also quickly developed in some patients^27^. Early prognosis of COVID-19 patients may aid in their specialized medical care reducing mortality.

The Diagnosis and Treatment Program of 2019 novel Coronavirus Pneumonia (trial version seven) categorizes COVID-19 patients into mild, moderate, severe, and critical^24^. It is now clear that COVID-19 mortality rate is the highest in elderly patients and the severe cases have reportedly elevated levels of some specific clinical parameters including WBC, D-dimer or lymphopenia. Current studies on COVID-19 have focused on its epidemiology and clinical features of the patients^28^, but fewer reported a specific prognostic marker. This study aimed to develop a model that precisely predicts the worst outcomes of the disease for the patients with COVID-19.

We analyzed the laboratory data as well as comorbidities and initial symptoms of 63 patients diagnosed with COVID-19 at Huoshenshan Hospital, Wuhan. All of the patients had developed severe disease condition resulting in in-hospital death of 46. Out of 63, 17 patients recovered and were discharged. As compared to the recovered patients, the laboratory parameters of deceased patients showed multiple organ damage including liver, kidney and heart, in addition to lungs. We attempted to evaluate high resolution biomarkers which would rapidly and efficiently sort COVID-19 patients in early stage of infection. The proper early diagnosis would help the medics in identifying severe cases for special treatment thus sparing resources for mild ones.

## Results

### COVID-19 is linked with sex and age

A total of 63 adult patients were included in the study. The mean age of the patients was 70 (range 50–80) as shown in Table 1. There was an unequal proportion of men (39) and women (24). The mean age of female patients was 72 (55–89), while 69 (50–86) in males. In the deceased patients group, this trend (aged and male were more vulnerable) is more obvious.

**Table 1:** Details of COVID-19 patients’ age and sex.

Patients	Overall	Deceased	Recovered
No. of patients	63	46	17
Male	39	30	9
Female	24	16	8
Average age	70.2 (50–80)	69.5 (50–88)	71.1 (52–89)
Average age of male	69.3 (50–86)	69.1 (50–88)	69.8 (52–84)
Average age of female	71.8 (55–89)	71.4 (63–88)	72.6 (55–89)

### Initial symptoms, chest CT scan and past medical history of COVID-19 patients

All 63 severe patients were hospitalized at the onset of COVID-19 signs and symptoms (Table 2), and later on confirmed with standardized protocols. Around 70% of patients had fever (low/high) while half of them had cough (51%) and fatigue (49%). Among them, 44% had breathing difficulty and 11% felt muscle pain all over the body. Their treatment duration averaged around 20 days with a minimum of 1 day and a maximum of two months during or after which they were discharged in case of in-hospital death or recovery, respectively. Apart from the nucleic acid test, the chest CT-scan showed infection in the lungs of all COVID-19 patients. CT-scan results showed either diffused patchy shadows or inflammatory lesions in lungs at the initial days of hospitalization of deceased as well as recovered patients.

**Table 2:** Initial symptoms, comorbidities and CT-scan results of COVID-19 patients.

Patients	Avg. days in hospital	Initial symptoms and CT-scan Results	Freq (%)	Comorbidities	Freq (%)
**Overall**	20.28			Hypertension	65
				Diabetes	22
		Multiple large plate-like ground-glass changes in lungs	100	Chronic Bronchitis	8
				Atherosclerosis	8
		Fever	68	Hypoproteinemia	8
		Cough	51	Anemia	8
		Fatigue	49	Uremia	5
		Breathing difficulty	44	Cerebral Infarction	5
		Muscle pain	11	Alzheimer's Disease	3
		Chills	11	Dementia	3
		Headache	3	Malignant tumor of esophagus	2
		Vomit	2	Emphysema	2
		Nausea	2	Epilepsy	2
				Hypoproteinemia	2
				Liver damage	2
**Deceased**	16.89	Multiple large plate-like ground-glass changes in lungs	100		
				Hypertension	67
		Fever	76	Diabetes	24
		Fatigue	61	Atherosclerosis	11
		Breathing difficulty	52	Hypoproteinemia	11
		Cough	50	Anemia	11
		Chills	15	Chronic Bronchitis	9
		Muscle pain	11	Uremia	7
		Headache	4	Cerebral Infarction	7
		Vomit	2	Alzheimer's Disease	4
		Nausea	2		
**Recovered**	31.42	Multiple large plate-like ground-glass changes in lungs	100	Hypertension	59
				Diabetes	18
		Cough	53	Dementia	12
		Fever	47	Malignant tumor of esophagus	6
		Breathing Difficulty	24	Chronic Bronchitis	6
		Fatigue	18	Emphysema	6
		Muscle pain	12	Epilepsy	6
				Hypoproteinemia	6
				Liver damage	6

In deceased patients, the initial symptoms included fever in 76% (P = 0.043), fatigue in 61% (P = 0.54), breathing difficulty in 52% (P = 0.45), cough in 50% (P = 0.615) while chills in 7% (P = 0.14) and muscle pain in 11% (P = 0.43) of the studied patients. These symptoms were not occurring altogether in an individual patient but randomly in mulitple patients, with fever and cough being the most common symptoms. Their average treatment duration starting from admission to death was around 17 days with a maximum of 50 days and minimum of 1. All of 46 deceased patients developed severe symptoms including ARDS.

On the other hand, in the recovered patients group, the average duration spent at hospital was around 1 month with a minimum of 1 week and a maximum of 2 months of treatment. They also displayed the same initial symptoms as half of them (~ 50%) had cough and an equal number of patients had fever, while 24% and 18% had breathing difficulty and fatigue, respectively.

As reported by many, COVID-19 infection also depends on the underlying health condition of the infected person. Patient with comorbidities such as hypertension, diabetes or others, would probably develop more severe disease condition. In order to evaluate their disease severity, we recorded the past medical history of all patents and noticed that 65% were suffering from hypertension, and 22% had diabetes. Several clinical conditions including chronic bronchitis, atherosclerosis, anemia and uremia were also present in some patients (Table 2).

### SARS-Cov-2 causes multi-organ damage

We know that the S (Spike) protein of the SARS-CoV-2 strongly binds to the receptors of ACE2^29^. ACE2 protein is expressed in almost all organs and tissues including colon, liver, intestinal epithelia, kidney, heart as well as ovaries and testes^30,31^. Due to this broad distribution, SARS-CoV-2 can target any organ/tissue leading to multi-organ failure^32^. Laboratory evaluation of COVID-19 patients demonstrated abnormalities in biomarkers of organs besides lungs. We noticed rise/fall in the level of several important biomarkers including cardiac, renal, hepatic, coagulatory and hemolymphatic.

### SARS-CoV2 distorts hemolymphatic balance

**Figure 1:**
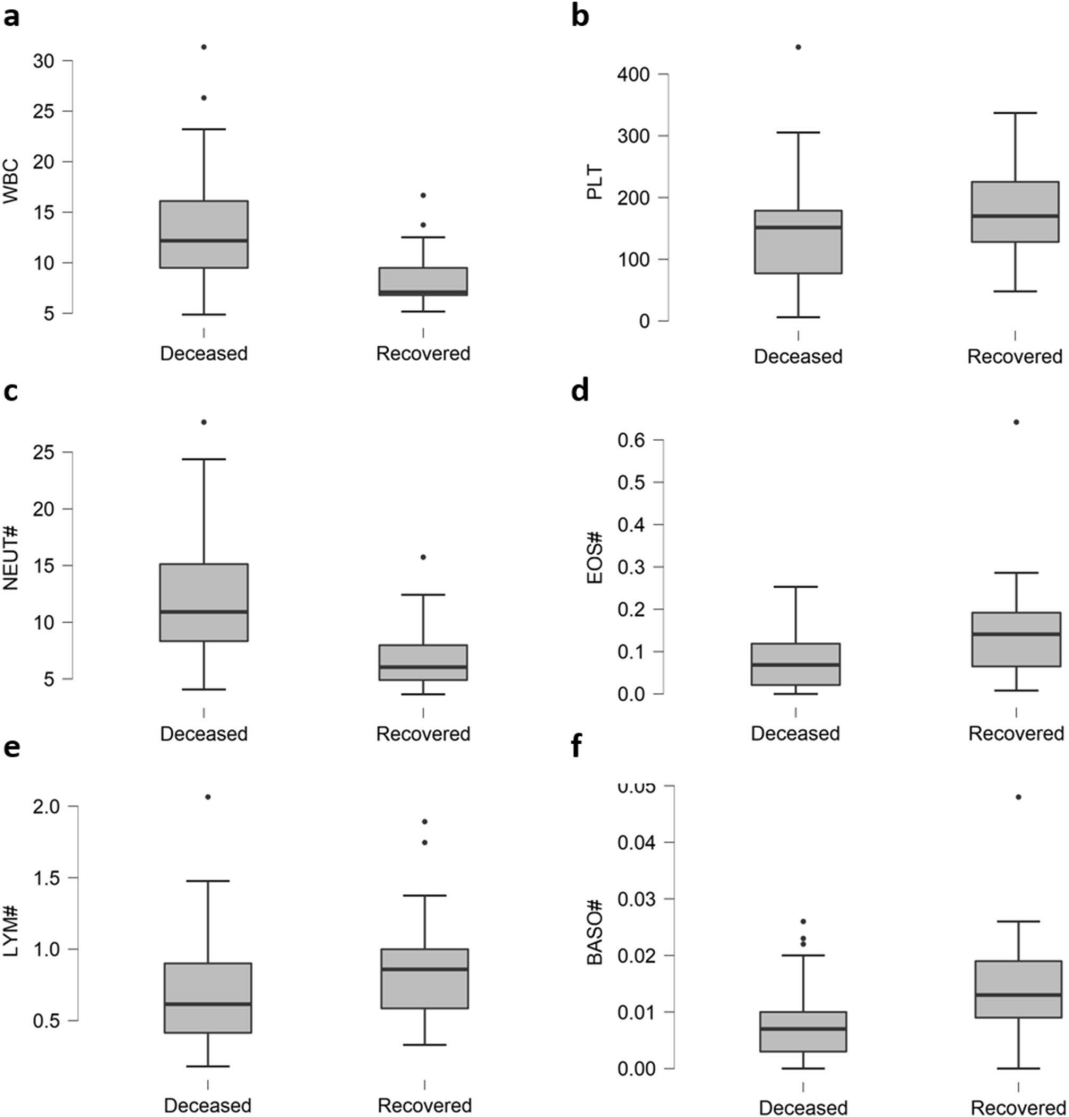
Boxplots depicting comparison of 6 hemolymphatic parameters of deceased and recovered patients. Elevated levels of WBC (**a**), NEUT# (**c**), LYM# (**e**), while decreased levels of PLT (**b**), EOS# (**d**) and BASO# (**f**) is common in deceased patients.

Majority of the deceased patients had imbalances in blood and lymphatic cells (Table 3). Among them, the level of some hemolymphatic parameters was significantly increased while that of others had dropped as shown in Fig. 1. Of 47 deceased patients, WBC (Fig. 1a) and NEUT (Fig. 1c) counts were elevated in 73% (P = 0.002) and 78% (P < 0.001), respectively. However, Platelets (Fig. 1b), EOS (Fig. 1d), LYM (Fig. 1e) and BASO (Fig. 1f) were decreased in 35% (P = 0.174), 46% (P = 0.004), 74% (P < 0.056) and 72% (P = 0.009) respectively, in deceased patients.

**Table 3:** Blood chemistry and frequency of rise/fall of biomarkers of 63 critical patients.

Marker			P value	Marker			P value
	Deceased (%)	Recovered (%)			Deceased (%)	Recovered (%)	
**Cardiac and sepsis**				**Hepatic**			
BNP > 125 pg/mL	50	41	0.787	ALT > 33 IU/L	74	41	0.135
Hs Trp I > 0.04 ng/mL	65	24	0.273	AST > 56 U/L	46	24	0.022
Myoglobin > 90 ng/mL	52	18	0.01*	TP < 60 g/L	83	65	0.6
PCT > 1 ng/mL	48	6	0.008*	GLB < 23 g/L	37	29	0.553
**Coagulatory**				A/G < 0.5	9	0	0.655
APTT > 40 s	7	0	0.031	ALP > 140 IU/L	15	12	0.223
D-Dimer > 1 mg/L	80	76	0.171	GLU > 7.1 mM/L	78	35	0.002
FIB > 4 g/L	26	18	0.635	LDH > 333 IU/L	85	24	< 0.001*
PT > 13.5 s	87	65	< 0.001*	HBDH > 280 U/L	76	24	< 0.001*
**Hemolymphatic**				CRP > 100 mg/L	65	6	< 0.001*
EOS# > 0.05 × 109/L	46	24	0.004*	**Renal**			
LYM# < 0.9 × 109/L	74	65	0.056	Urea > 7.1 mM/L	74	41	0.014*
MONO# < 0.3 × 109/L	30	6	0.784	Cre > 107 mM/L	37	12	0.102
NEUT# > 7.5 × 109/L	78	35	< 0.001	CysC > 1.10 mg/L	74	65	0.384
PLT < 150 × 109/L	48	35	0.174	CO2 < 23 mEq/L	54	18	0.003
WBC > 10.5	74	24	0.002	CK > 300 U/L	15	0	0.002*

Deceased (D), Recovered (R), B-Type Natriuretic Peptide (BNP), Hypersensitive Troponin I (Hs Trp I), Procalcitonin (PCT), Activated Partial Thromboplastin Time (APTT), Fibrinogen (FIB), Pro-Thrombin Time (PT), Eosinophil (EOS), Lymphocyte (LYM), Monocytes (MONO), Neutrophil (NEUT), Platelet (PLT), White Blood Cell Count (WBC), Alanine Aminotransferase (ALT), Aspartate Aminotransferase (AST), Total Protein (TP), Globulin (GLB), Albumin/Globulin Ratio (A/G), Glucose (GLU), Lactic Acid Dehydrogenase (LDH), α-Hydroxybutyrate Dehydrogenase (HBDH), C-Reactive Protein (CRP), Uric Acid (UA), Creatinine (Cre), Cystatin C (Cysc), Creatine Kinase (CK).

* Levene’s test is significant (p < 0.05), suggesting a violation of the equal variance assumption.

### Hepatic impairments

**Figure 2:**
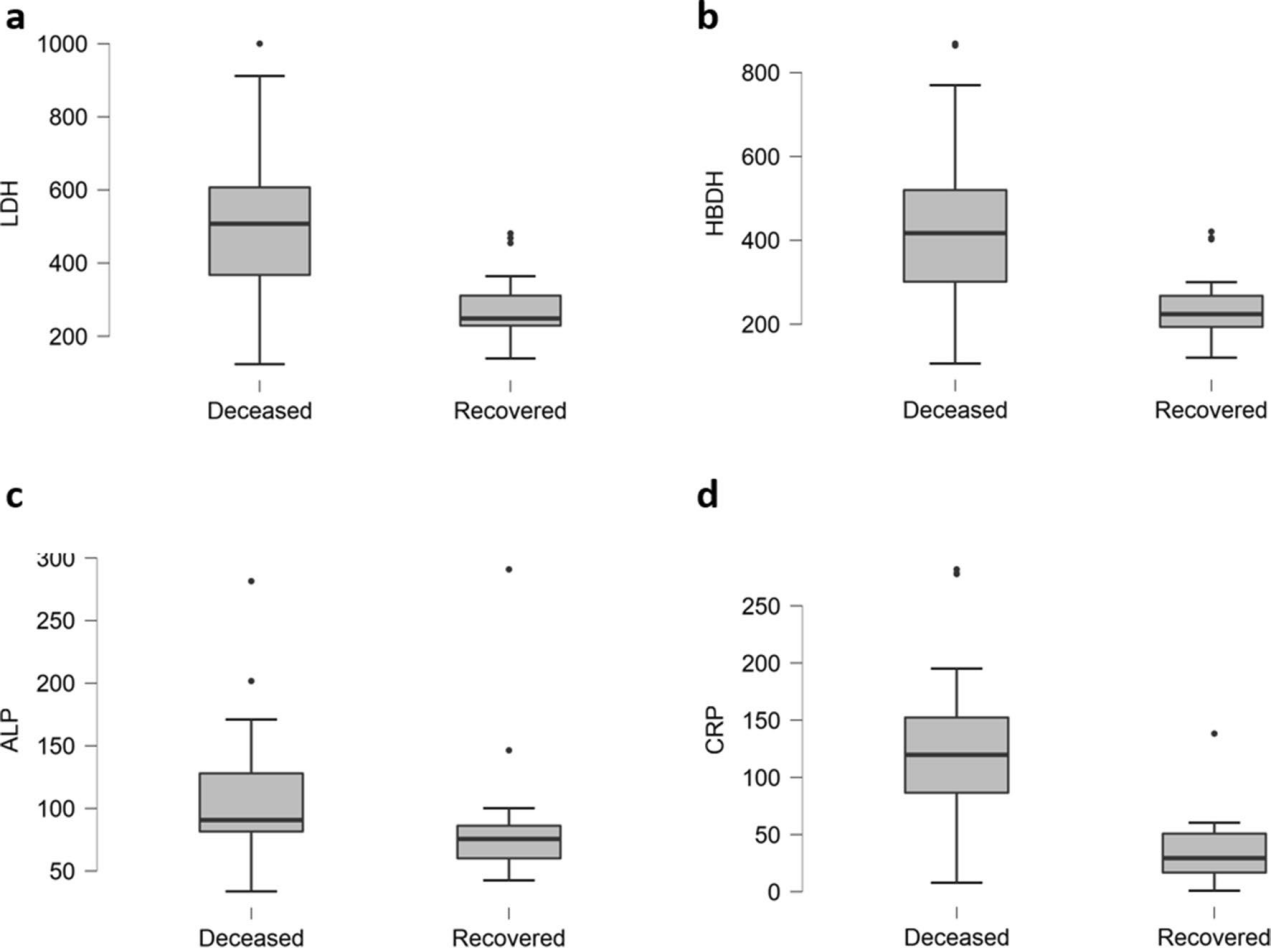
Hepatic biomarkers of deceased and recovered patients As compared to recovered patients, elevated levels of LDH (**a**), HBDB (**b**), ALP (**c**) and CRP (**d**) could be observed in deceased patients.

A significant rise in the liver enzyme AST and ALT (Alanine aminotransferase) is usually linked with hepatic impairments^8^. We noticed serious liver damage in a major portion of deceased patients. As shown in Fig. 2, all four predictors including LDH (85%, P < 0.001, Fig. 2a), HBDH (76%, P < 0.001, Fig. 2b), ALP (15%, P = 0.223, Fig. 2c) and CRP (65%, P < 0.001, Fig. 2d) were significantly elevated in deceased patients. Besides, ALT and AST increased in 74% (P < 0.001) and 46% (P < 0.001) of deceased patients, respectively. In contrast, TP decreased in 83% (P = 0.142) and GLB in 37% (P = 0.619) in deceased patients as mentioned in Table 3 and File S1.

### Renal damage

**Figure 3:**
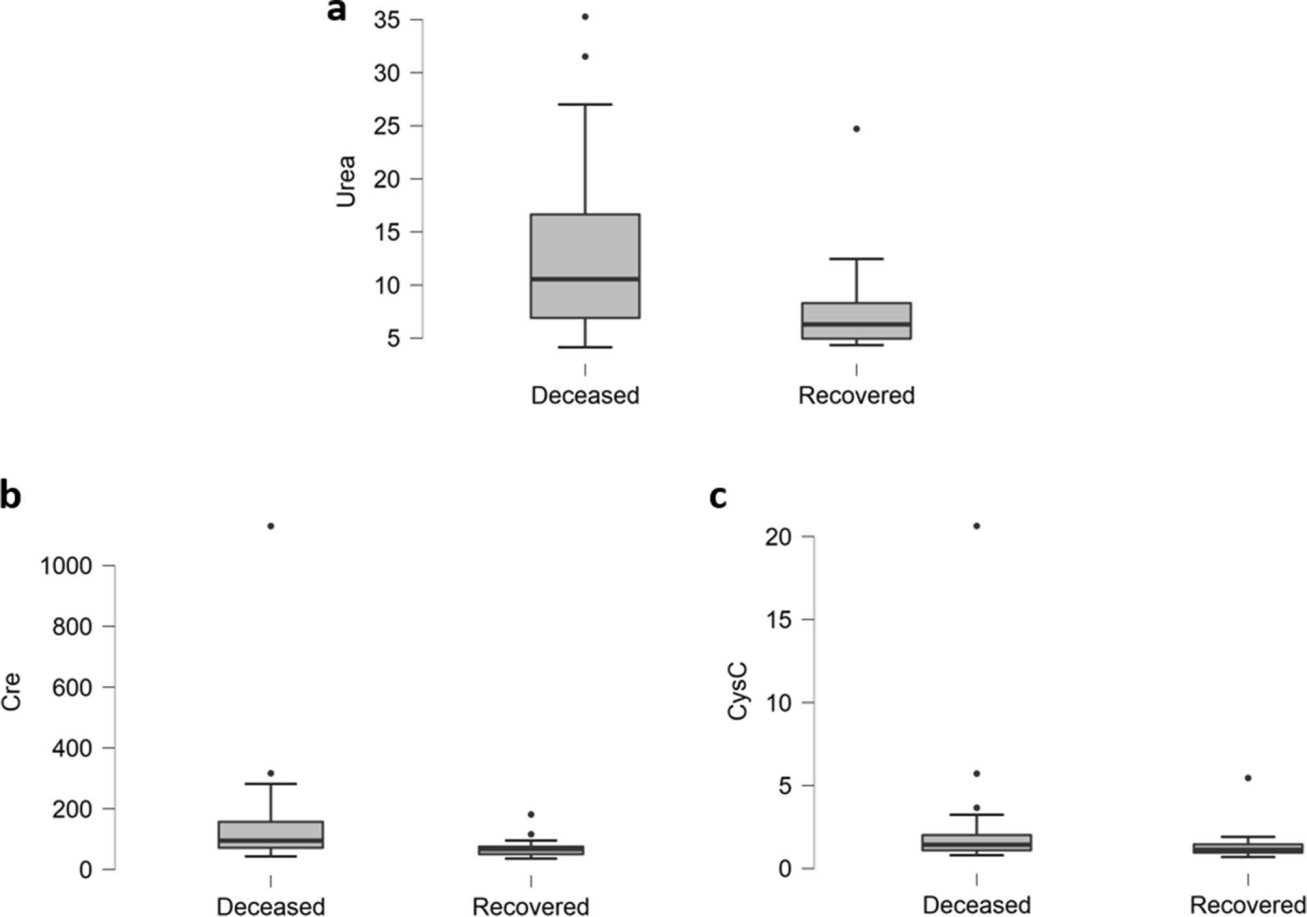
Comparison of renal biomarkers of deceased and recovered patients. (**a**) Urea is significantly increased, while Creatinine (**b**) and Cystatin C (**c**) are slightly elevated in deceased patients.

In deceased patients, renal damage was obvious from the elevated levels of urea (Fig. 3a), Creatinine (Fig. 3b) and CystatinC (Fig. 3c). As depicted in Fig. 3, Urea (74%, P = 0.014), Cre (37%, P = 0.102) and CysC was increased in 74% (P = 0.384) of deceased patients. Therefore, renal abnormalities also played significant role in the progression of disease and contributed to overall damage caused by SARS-CoV-2 leading to death.

### Cardiac injury and sepsis

**Figure 4:**
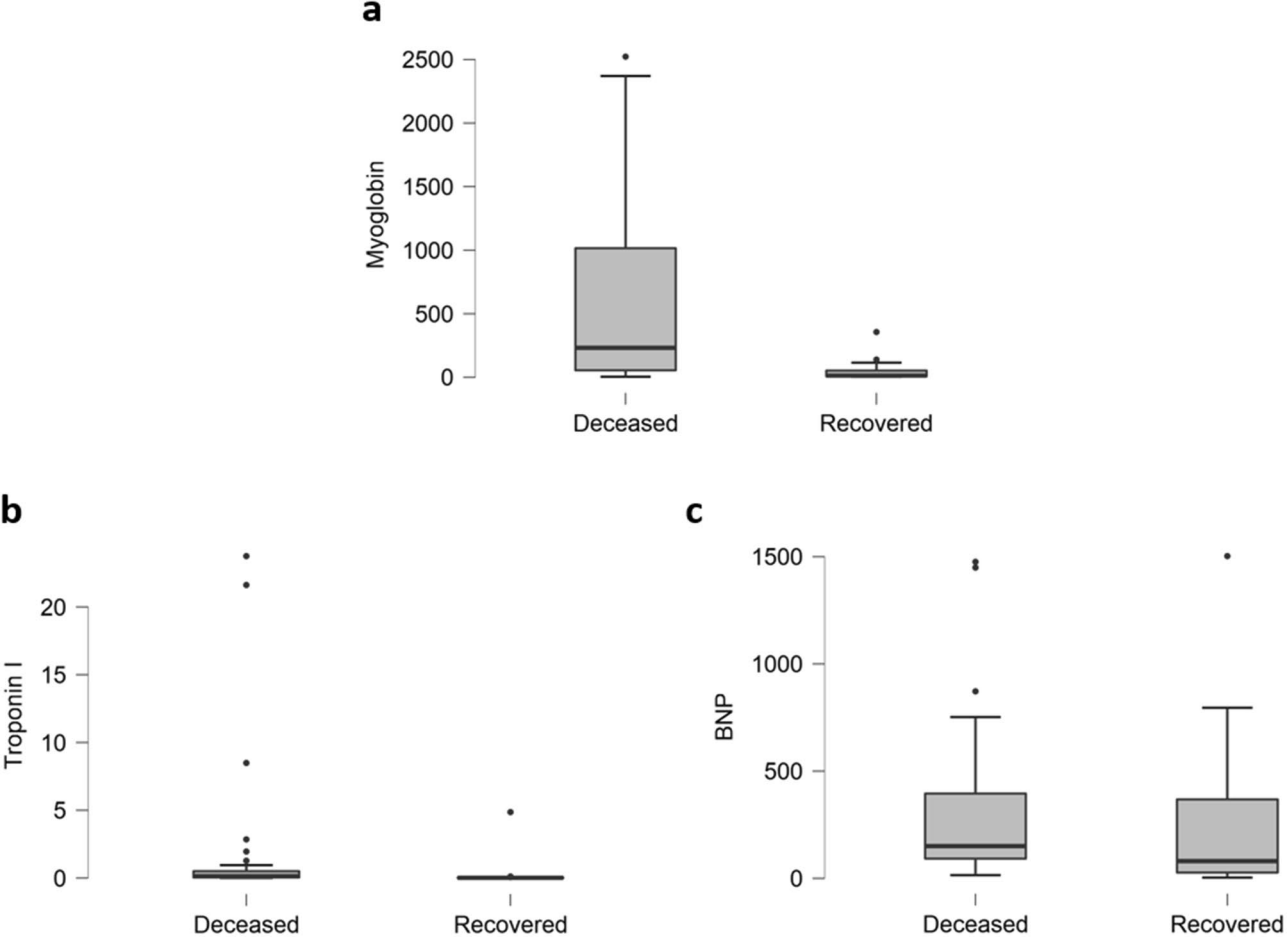
Cardiac parameters of deceased and recovered patients. Highly elevated levels of (**a**) Myoglobin, while slight elevations in Cardiac Troponin I (**b**) and B-Type Natriuretic Peptide-BNP (**c**) have occurred in deceased patients.

Cardiac damage can be noticed from significant elevation in the level of Myoglobin, Cardiac Troponin and B-Type Natriuretic Peptide (BNP). Elevated level of Myoglobin (52%, P < 0.01, Fig. 4a), Troponin-I (65%, P = 0.27, Fig. 4b) and BNP (50%, P = 0.787, Fig. 4c) are obvious (Fig. 4 and Table 3).

### Coagulatory imbalances

**Figure 5:**
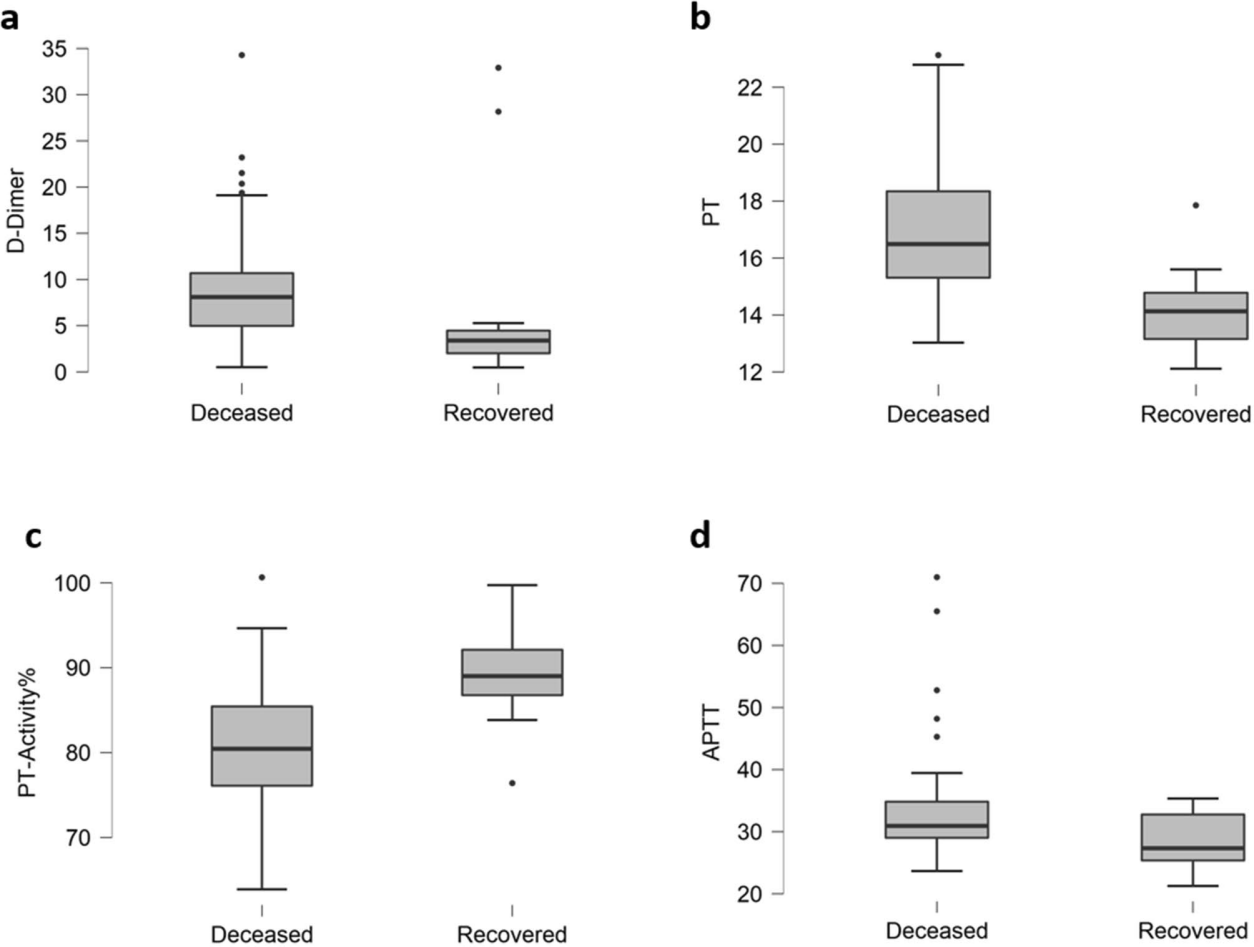
Coagulatory parameters of deceased and recovered patients. Elevations in (**a**) D-dimer, (**b**) PT and (**d**) and APTT while decline in (**c**) PT-Activity% can be seen in deceased patients.

In deceased patients, high levels of FDP and D-dimer (fibrin-associated markers) points towards a usual activation of coagulatory agents and secondary fibrinolysis. Our results support the previous findings^33^, as we noticed imbalances of coagulatory indicators more pronounced in deceased patients (Fig. 5). There were elevations in the level of D-dimer (80%, P < 0.171, Fig. 5a), PT (87%, P = 0.031, Fig. 5b) and APTT (57%, P = 0.053, Fig. 5d), while PT-Activity% (52%, P < 0.001, Fig. 5c) was declined in deceased patients. Increased level of Fibrinogen (26%, P = 0.635) was also observed in deceased patients, as given in Table 3.

### Significant laboratory parameters for early discrimination of COVID-19 patients

**Figure 6:**
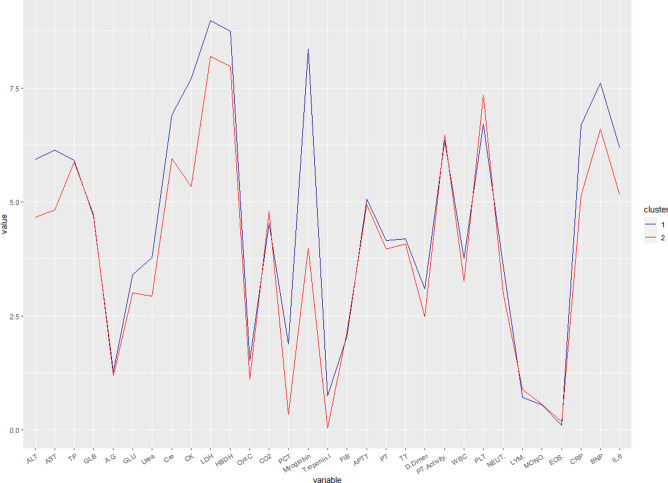
Cluster analysis of significant clinical parameters in deceased and recovered patients. Blue line (1) indicates dead while red (2) indicates recovered patients.

From our analysis of 63 severe patients, we observed prominent differences in nine parameters (Fig. 6). They span cardiac, hepatic and hemolymphatic biomarkers, supporting the evidence that SARS-CoV2 affects multiple organs in vulnerable individuals. Of cardiac biomarkers, Myoglobin specifically could efficiently predict COVID-19 severity in its early stages. Similarly, among the coagulatory parameters, elevated d-dimer level seems to be an efficient early predictor of COVID-19 severity. Likewise, elevated levels of CRP, LDH and HBDH could be considered as some of the high resolution hepatic biomarkers for early prognosis. SARS-CoV-2 infection is marked by lymphopenia and abnormal cardiac indicators, all of which hold significant prognostic value, however, the underlying mechanisms still need to be studied in detail. Elevated levels of WBC and Eosinophils, while lymphopenia are more common in severe cases who succumbed to COVID-19 and hence should be included among the other early predictors.

## Discussion

Globally, public heath poses high risk of emerging and reemerging pathogens^34^. Coronaviruses are among the widely distributed RNA viruses causing various diseases including hepatic, enteric, neurologic and respiratory disorders in humans as well as other animals. In the present study, the average age of 70 years shows that elderly people are more vulnerable to SARS-CoV2 severe infection, as highly cited in the literature. The unequal ratio of males and females 63% and 38% respectively, shows that males are more targeted, which is in accordance with previous reports^35^. In our study, the median length for hospital stay was about 20 days while the overall length matched to two previous studies of 11 and 12 days^36^. Similarly, the underlying medical conditions of COVID-19 patients were mostly the same as those with critical infectious diseases demanding ICU or hospital admission^37^. Chronic obstructive pulmonary disease and asthma could not be seen as risk factors for severe COVID-19 cases^38^. This has no known epidemiologic reasons. Although, comorbidities as well as age were two main predictors for hospital admission, they could not prove to be effective prognostic indicators. In contrast, oxygen impairment and inflammatory markers were marked by severe disease condition and even mortality^2^^.^ In our study, the most common symptoms were the same as reported previously^39^, including fever, cough, fatigue, breathing difficulty and muscle pain.

CT-scan results showed infection of both lungs in all patients proving the lungs as the main target of SARS-CoV2. COVID-19 disease also depends on the underlying health condition of the infected patient. In order to evaluate their disease severity, we recorded the past medical history of all patients. Majority of patients suffered from hypertension and diabetes while some also had atherosclerosis, Dementia, Alzheimer’s disease, hypoproteinemia and liver damage. Patients with underlying medical conditions are more vulnerable to COVID-19 and would probably develop severe disease condition leading to death. Such patients should be prioritized for focused care considering their past medical history.

Acute respiratory infections are well-recognized triggers for cardiovascular diseases (CVD), and the underlying CVD is usually associated with comorbidities, which may increase the incidence and severity of infectious diseases. SARS-CoV2 targets ACE2 receptor, the role and significance of which has not yet been fully unraveled, however, its elevated level has been noticed in a number of chronic diseases including diabetes, hypertension, chronic kidney disease and others^40,41^. The substrates of ACE2 span aplin, ghrelin, kinins, neurotensin, angiotension and dynorphin^42^. ACE2 mainly regulates angiotensin II by converting it into angiotension 1–7 (which protects tissues), thereby physiologically counterbalancing ACE^31^. Heart and blood vessels are rich in ACE-2 (the target of SARS-CoV2). The presence of cardiac injury, myocarditis and ARDS are other strong and independent factors associated with morality^43^. Heart damage and arrhythmias are common in patients hospitalized for COVID-19^44^. The rise and/or fall of troponin indicating myocardial injury is common among patients with acute respiratory infections and correlated with disease severity^45^. Abnormal troponin values are common among COVID-19 patients particularly evaluated though a high sensitivity cardiac troponin assay^45^. Abnormal blood clotting (causing pulmonary embolism or stroke) and blood vessel constriction have also been reported previously^46^. Adjunctive cardioprotective therapies may be advisable in patients with significant elevation of cardiac injury biomarkers. Initial measurement of cardiac biomarkers immediately after hospitalization for SARS-CoV2 infection may help identify a subset of possible cardiac injury.

Earlier, similar findings were reported in the deceased COVID-19 patients with higher levels of hemolymphatic indices such as TB (total bilirubin), WBC , CK as well as IL-6^12,47,48^. Lymphopenia was reported in 80%^12^ of severe and 25%^11^ of mild cases in two independent studies, correlating lymphopenia to critical condition. Renal damage was found in 78% out of 33.9% COVID-19 patients while recently studying 1000 COVID-19 patients^1^. However, acute kidney damage was developed by 15%^49^ and 19%^50^ of COVID-19 patients in two independent studies. D-dimer is a biomarker of pathological coagulation that underlies the pathogenesis of most cardiovascular diseases^51^. The values of D-dimer are frequently increased in severe cases with in-hospital deaths hence its measurement may be helpful in predicting evolution of COVID-19 disease towards a worse clinical picture^51,52^. D-dimer may help to define whether adjunctive antithrombotic therapies (e.g., anticoagulants, antithrombin or thrombodulin) might be helpful with severe COVID-19.

CRP is routinely used as a non-specific marker of inflammation. CRP does not normally elevate significantly in mild viral respiratory infection however, significant increase of CRP has been reported in COVID-19 patients^16,53^. CRP testing may be useful in the initial evaluation of COVID-19 patients. A study of 140 COVID-19 patients found elevated levels of IL-6 in 95, CRP in 91 and PCT in 8 patients, at admission. They found CRP > 41.8 mg/L in severe cases and suggested that the elevated levels of CRP and IL-6 could efficiently predict respiratory deterioration^54^. Similarly, in a meta-analysis, Sahu et al., compared the CRP levels of 849 deceased and 1896 recovered COVID-19 patients. They found high concentrations of CRP in deceased patients and suggested that CRP could efficiently predict COVID-19 disease prognosis^55^. A multicentered retrospective study involving 3219 patients also found significantly elevated levels of CRP in severe cases with in-hospital death^56^.

Several studies have associated elevated levels of myoglobin with COVID-19 severity^50,56^. SARS-Cov2 might induce a myositis similar to that observed in severe influenza infections. Rapid clinical recognition of muscle injury in COVID-19 patients can be lifesaving. Serum albumin (HSA) low levels are associated with increased risk of death^57^. Decreased level of HSA may be a medical sign of decreased production in the liver, increased loss in the gastrointestinal tract or kidneys, and/or increased use in the body. The combination of the hypo-albuminemia, lymphopenia, and high concentrations of CRP and LDH in SARS-CoV2-infected patients upon hospital admission may predict more severe acute lung injury^58^. A previous study including 288 severe and non-severe COVID-19 patients found myoglobin higher than 106 μg/L, WBC higher than 10 × 109/L, and CRP higher than 10 mg/L as risk factors for severe cases^59^. Similarly, another study of 357 deceased and recovered cases reported elevated levels of myoglobin along with CK-MB in patients resulting in in-hospital death^60^. However, an elevated level of myoglobin (≥ 306.5 μg/L) was found to be a risk factor for in-hospital death, independent of Troponin and CK-MB levels^61^.

Taking together, the previous studies support our findings and thus we report that CRP and myoglobin could prove efficient prognostic biomarkers for COVID-19 associated mortality.

## Conclusions

COVID-19 can be conspicuously phasic, with major deteriorations in some patients occurring ~ 7 d post-symptom onset. Patients with comorbidities specifically hypertension and diabetes mellitus, are particularly susceptible to COVID-19 infection and are likely to develop more severe illness. Furthermore, by the current clinical evaluation and statistical assessment, we identified myoglobin and CRP as specific risk factors related to organ failure and mortality. The identified risk factors should be specifically continuously screened for prognosis of COVID-19 patients in early stages.

## Materials and methods

### Ethical approval

The study design and protocol were reviewed and approved by Institutional Review Committee of Huoshenshan Hospital and Southern Theater Command General Hospital (approval number: 202013), and the requirement for informed consent was waived by the Ethics Commission. All methods were performed in accordance with the relevant guidelines and regulations.

### Study design and participants

This retrospective cohort study was executed in the Intensive Care Unit of Huoshenshan Hospital, Wuhan. Patients who were diagnosed with COVID-19 between 4th February 2020 and 12th March 2020, were screened. The data of all patients were divided into two sets. The first set consisted of patients who died of the infection during treatment in the hospital. The second set included those critical patients who recovered and were discharged from the hospital after two consecutive negative tests of COVID-19. Data regarding their age, sex, initial symptoms and past medical history were properly recorded.

### Initial symptoms, chest CT-scan and past medical history

The initial symptoms of all patients (deceased/recovered) admitted, were recorded. The collected data consisted of demographics, comorbidities, presenting symptoms, and laboratory and radiographical findings of the COVID-19 patients. The severity of COVID-19 was defined by satisfying at least one of the following factors; the ratio of the partial pressure of arterial oxygen (PaO2) to the fraction of inspired oxygen (FiO2) 300 mmHg (1 mmHg ¼ 0.133 kPa) breathing rate > 30/min and pulse oximeter oxygen saturation at rest < 93%. It was considered a critical illness if satisfying at least one of the following items, the respiratory failure occurred and individuals received mechanical ventilation, failure of other organs, shock, and received care in the intensive care unit.

### Blood chemistry analyses

In order to screen various hematologic and biochemical parameters including ALT, AST, CRP, D-dimer and others, their (deceased/recovered patients) blood samples were analyzed and the data of each patient was properly recorded. For inference, all laboratory parameters values were processed and their mean values were compared to the normal range values.

### Statistical analysis

The mean, median and inter quartile range (IQR) values were calculated as continuous variables while categorical variables were presented as frequencies and percentages of patients. Independent sample t-test or Mann–Whitney U test was used to compare distributions of continuous variables. Univariate and multivariate logistic regression analyses were executed to identify risk factors of disease progression. P < 0.05 was considered statistically significant.

## Supplementary Information

Supplementary Legends.

Supplementary Information.

## Abbreviations

BNP: B-type natriuretic peptide
Hs TrpI: Hypersensitive troponin I
PCT: Procalcitonin
APTT: Activated partial thromboplastin time
FIB: Fibrinogen
PT: Pro-thrombin time
EOS: Eosinophil
LYM: Lymphocyte
MONO: Monocytes
NEUT: Neutrophil
PLT: Platelet
WBC: White blood cell
ALT: Alanine aminotransferase
AST: Aspartate aminotransferase
TP: Total protein
GLB: Globulin
A/G: Albumin/globulin ratio
GLU: Glucose
LDH: Lactic acid dehydrogenase
HBDH: α-Hydroxybutyrate dehydrogenase
CRP: C-reactive protein
UA: Uric Acid
Cre: Creatinine
CysC: Cystatin C
CK: Creatine kinase
