# Supplementary material for: Myoglobin and C-reactive protein are efficient and reliable early predictors of COVID-19 associated mortality
Source: Sci Rep. 2021 Mar 16;11:5975. doi: 10.1038/s41598-021-85426-9 (PMC7971049; doi:10.1038/s41598-021-85426-9)
Supplement: Supplementary file 1 — Supplementary Legends. [file 41598_2021_85426_MOESM1_ESM.docx]

**Supplementary Materials**

**Figure S1**. Age, sex, comorbidities and major initial symptoms in all patients, **Figure S2**. Age, sex, comorbidities and major initial symptoms in deceased patients, **Figure S3**. Age, sex, comorbidities and major initial symptoms in recovered patients. **Appendix A:** Boxplots of the rest of laboratory parameters of deceased and recovered patients
