# Supplementary material for: Myoglobin and C-reactive protein are efficient and reliable early predictors of COVID-19 associated mortality
Source: Sci Rep. 2021 Mar 16;11:5975. doi: 10.1038/s41598-021-85426-9 (PMC7971049; doi:10.1038/s41598-021-85426-9)
Supplement: Supplementary file 2 — Supplementary Information. [file 41598_2021_85426_MOESM2_ESM.pdf]

Article

Myoglobin and C-Reactive Protein are Efficient and Reliable Early Predictors of COVID-19 Associated Mortality

Ashaq Ali[1,9#], Muhammad Noman[4#], Yong Guo[2,10#], Xu Liu[6#],Rong Zhang[5,8], Juan Zhou[1], Yang Zheng [7], Xian-En Zhang[7], Yong Qi[3*], Xiaohua Chen[2*], Dong Men[1,9*]

[1] State Key Laboratory of Virology, Wuhan Institute of Virology, Center for Biosafety Mega-Science, Chinese Academy of Sciences, Wuhan, 430071, China.

[2] Department of Laboratory Medicine, General Hospital of Central Theatre Command, PLA. Wuhan 430070, China.

[3] Medical Department General Hospital of Central Theatre Command, PLA, Wuhan 430070, China

[4] College of Life Science, Engineering Research Center of the Chinese Ministry of Education for Bioreactor and Pharmaceutical Development, Jilin Agricultural University, Changchun 130118, China.

[5] Department of Laboratory Medicine, General Hospital of Southern Theatre Command, PLA. Guangzhou 510010, China.

[6] Department of Biochemistry and Molecular Biology, Medical College, Hubei Minzu University, Enshi 445000, Hubei, China.

[7] National Laboratory of Biomacromolecules, CAS Center for Excellence in Biomacromolecules, Institute of Biophysics, Chinese Academy of Sciences, Beijing, 100101, China.

[8] Joint Expert Group for COVID-19, Wuhan Huoshenshan Hospital, Wuhan, Hubei 430100, China.

[9] University of Chinese Academy of Sciences, Beijing, 100049, China.

[10] Department of Pathology, General Hospital of Central Theatre Command, PLA. Wuhan 430070, China.

# Contributed equally

* Correspondence: qiy9527@163.com (Y.Q.); cxhniuniu@163.com (X.C.); d.men@wh.iov.cn (D.M.)

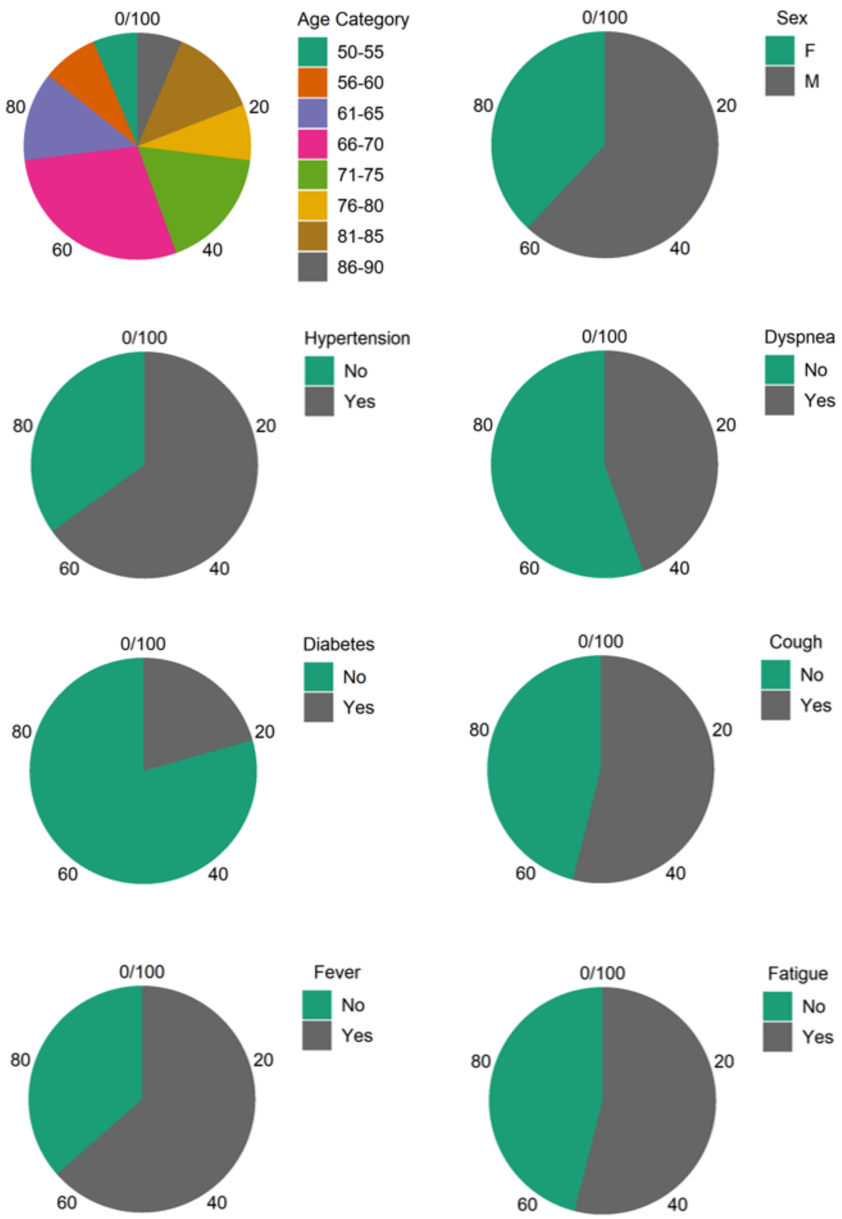

Figure S1: Age, sex, comorbidities and major initial symptoms in all patients

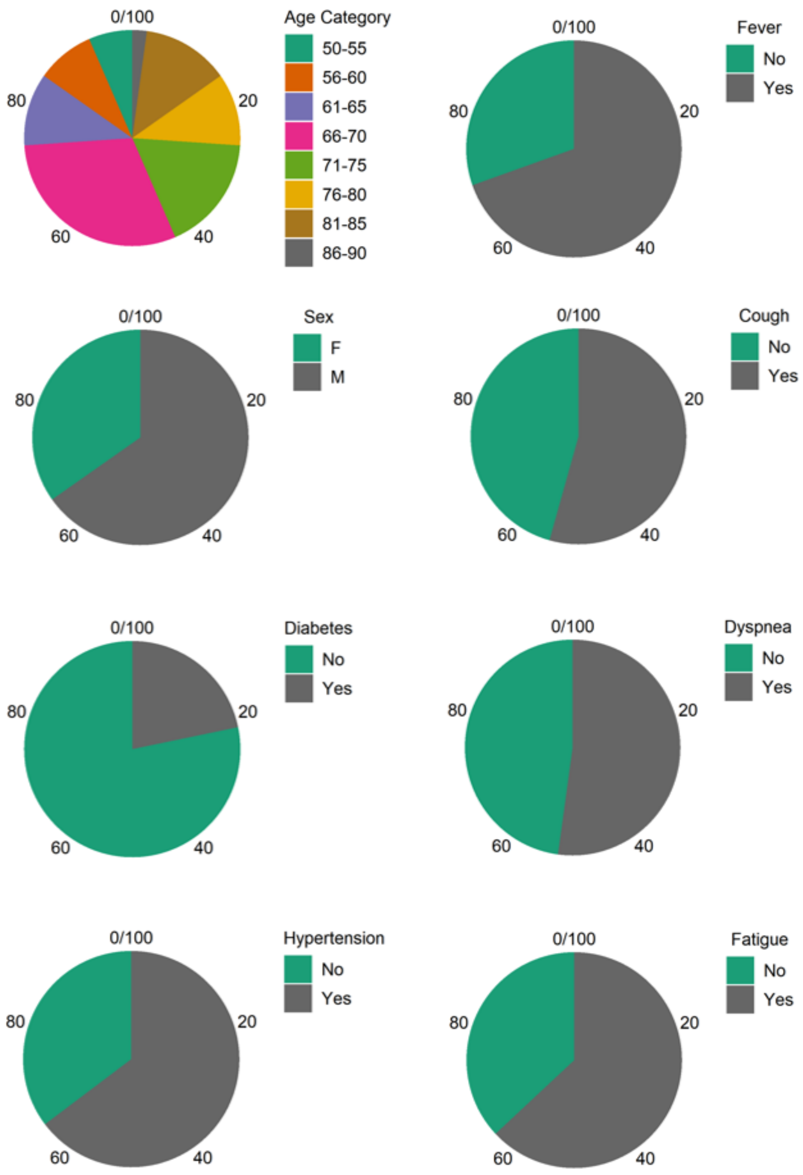

Figure S2: Age, sex, comorbidities and major initial symptoms in deceased patients

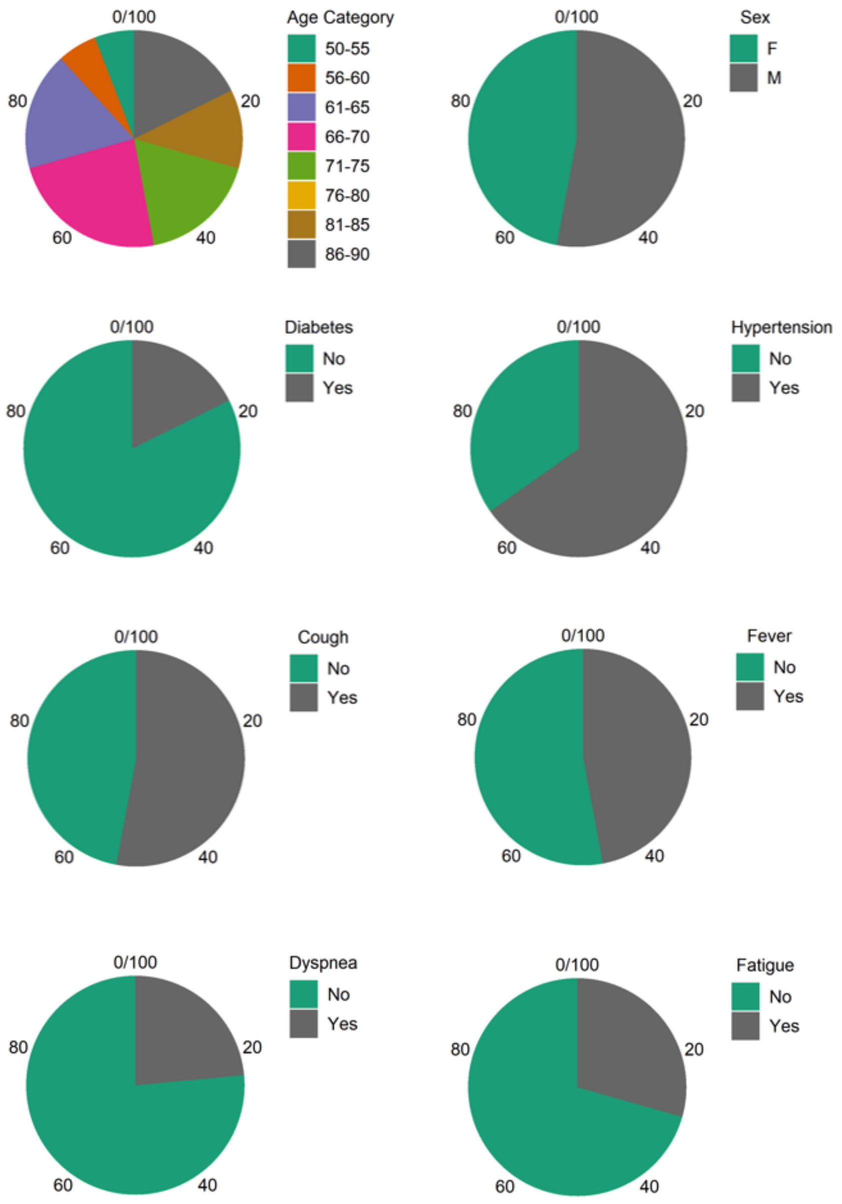

Figure S3: Age, sex, comorbidities and major initial symptoms in recovered patients

Appendix A: Boxplots of the rest of laboratory parameters

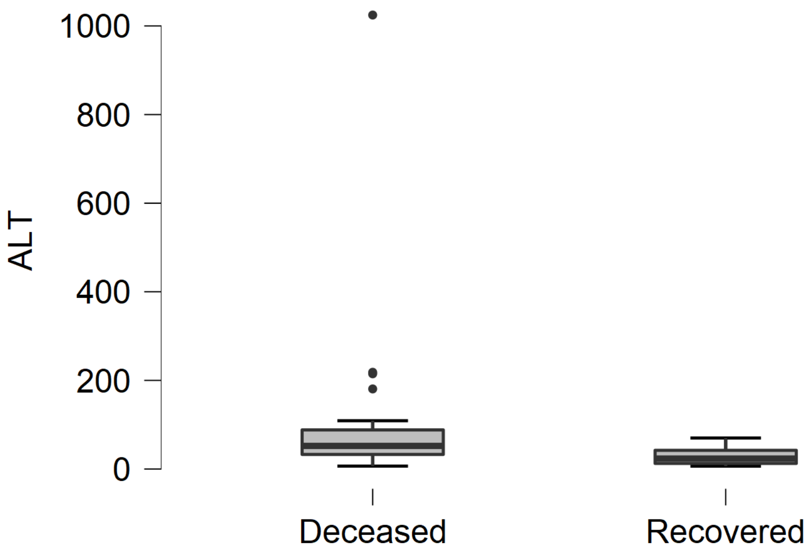

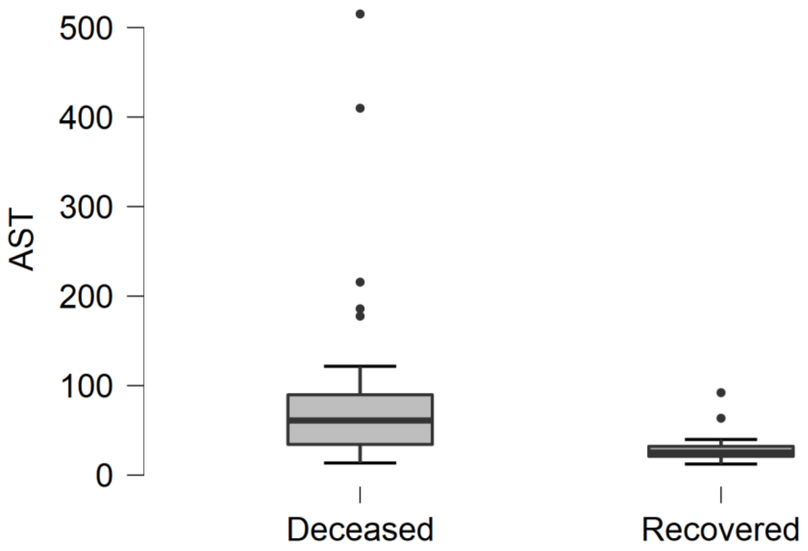

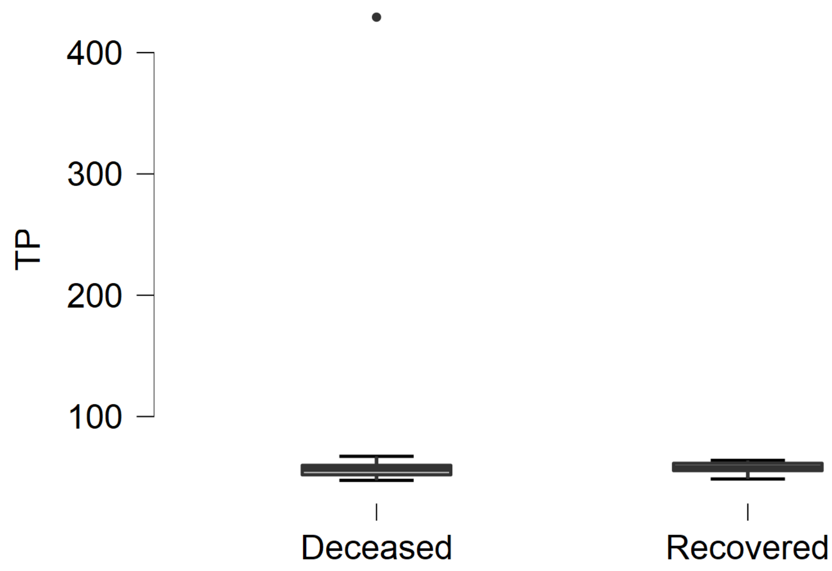
TP
400
300
200
100
Deceased
Recovered

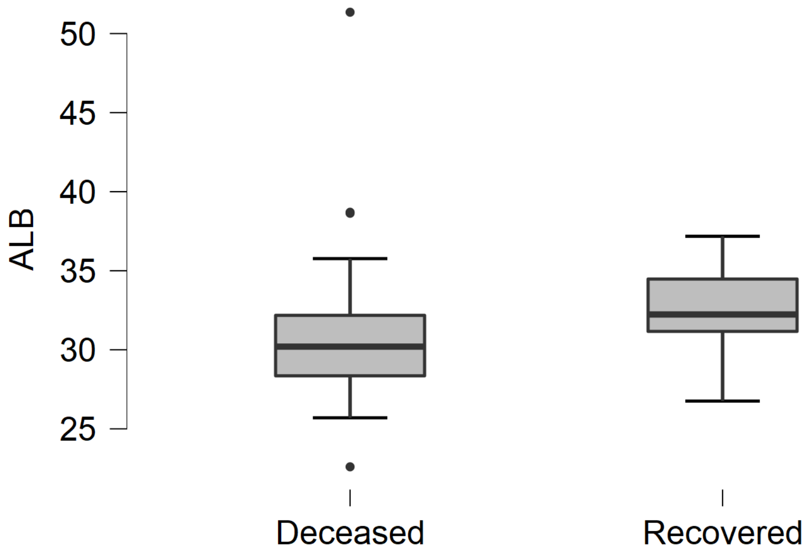
ALB
50
45
40
35
30
25
Deceased
Recovered

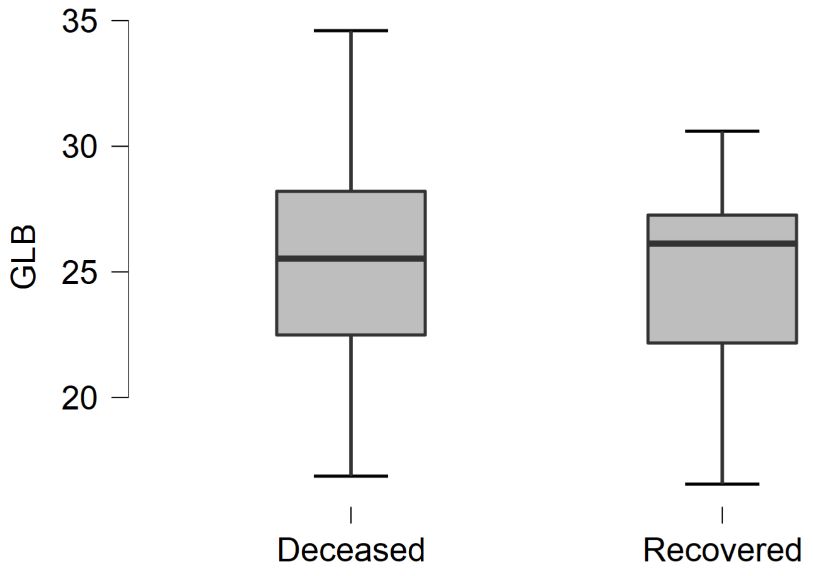
35
30
25
20
GLB
Deceased
Recovered

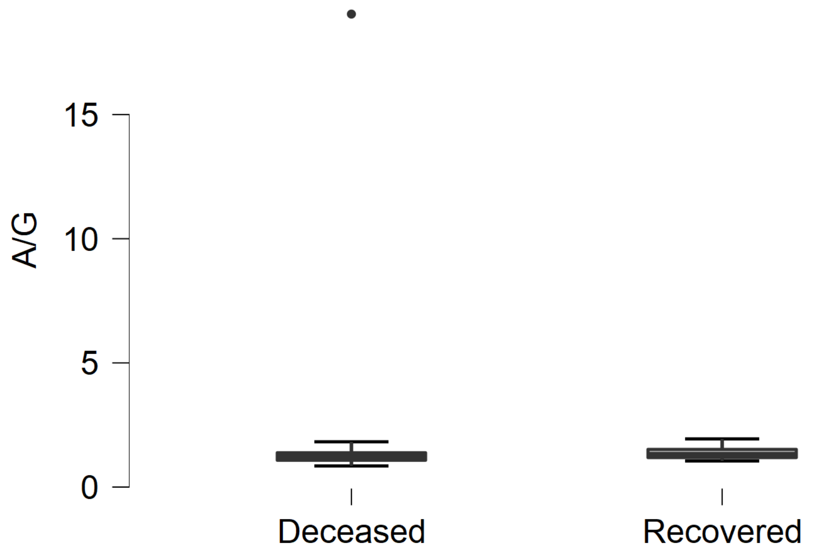
15
10
5
0
A/G
Deceased
Recovered

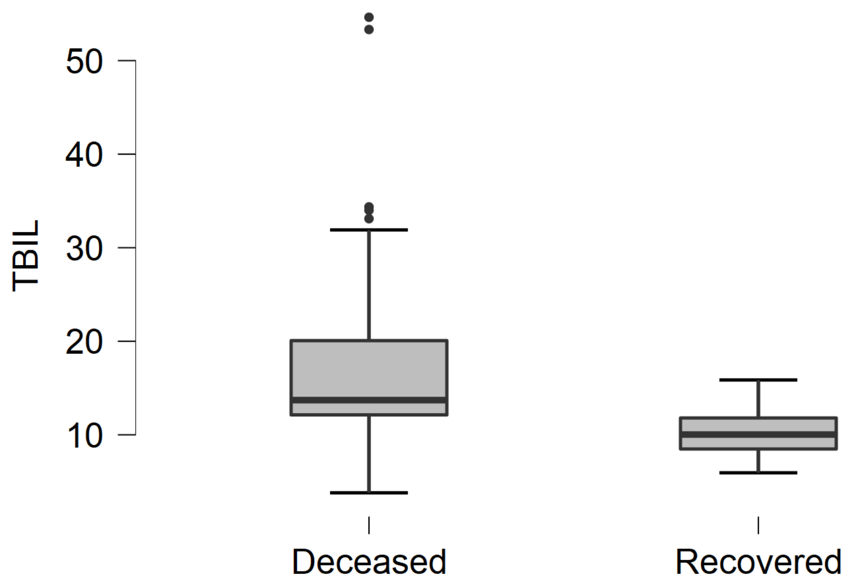
TBIL
50
40
30
20
10
Deceased
Recovered

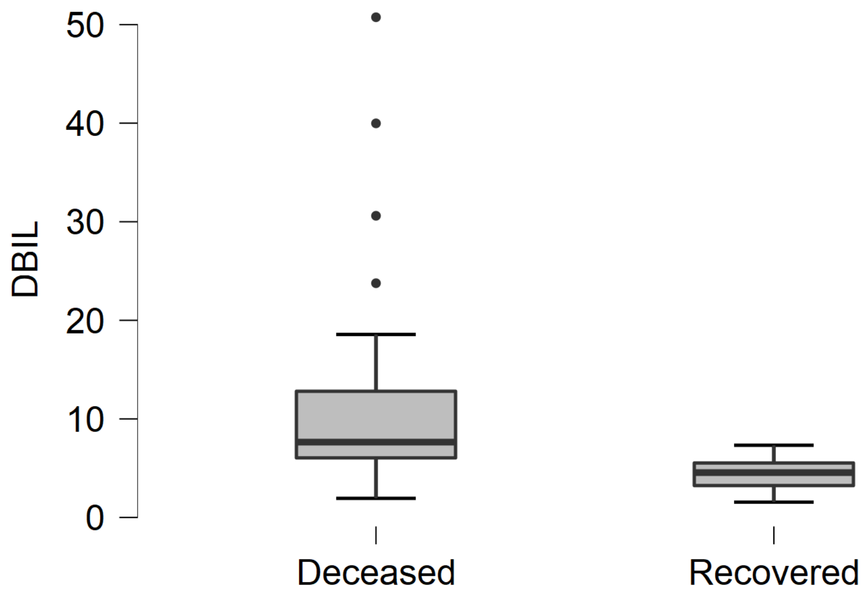
DBIL
50
40
30
20
10
0
Deceased
Recovered

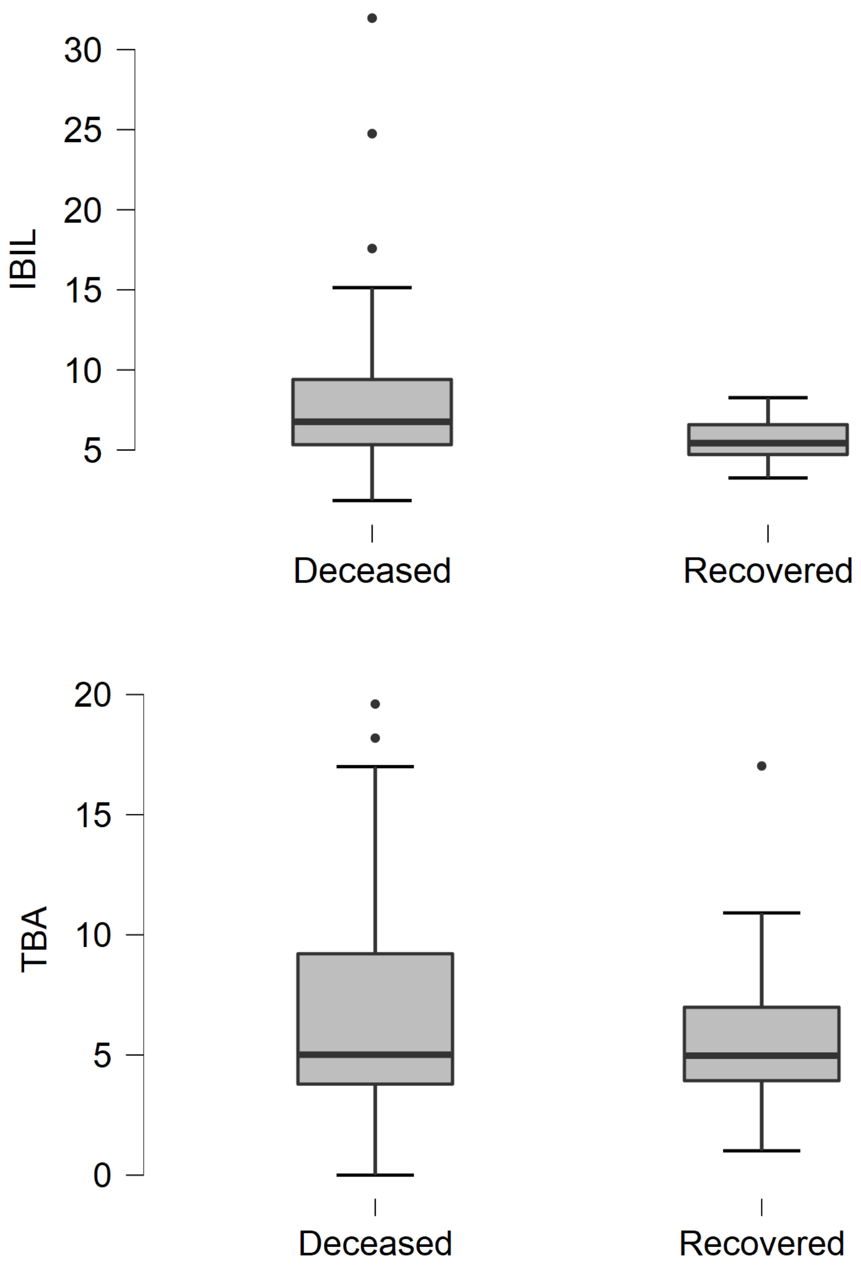
30
25
20
15
10
5
IBIL
Deceased
Recovered
20
15
10
5
0
TBA
Deceased
Recovered

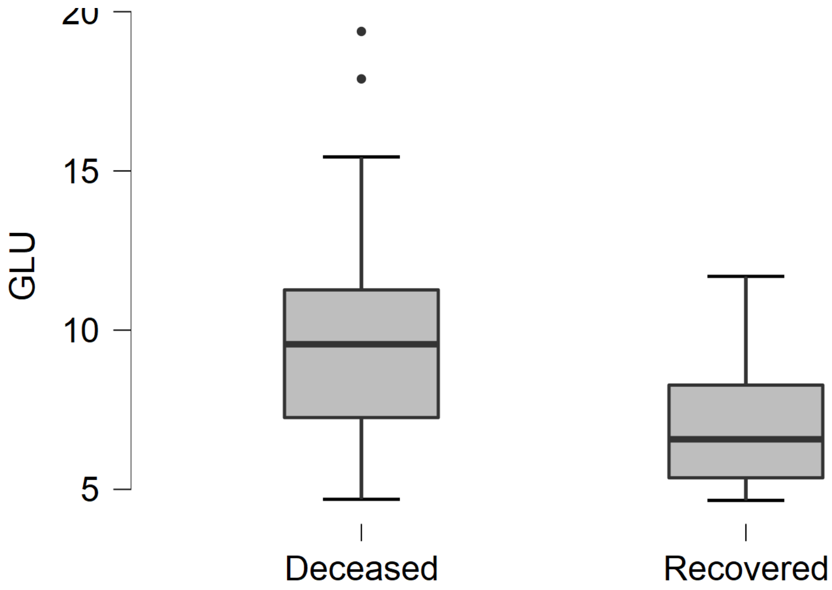
20
15
10
5
GLU
Deceased
Recovered

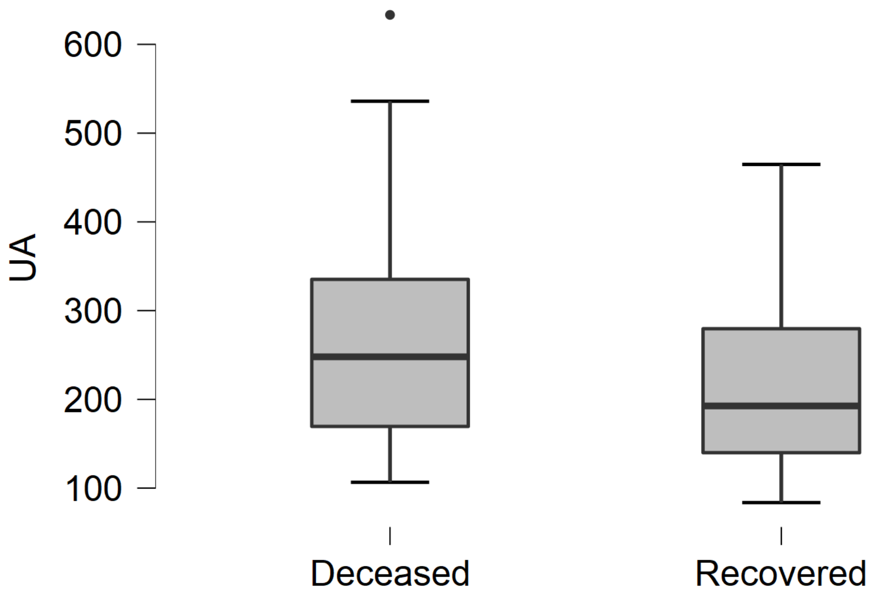
600
500
400
300
200
100
UA
Deceased
Recovered

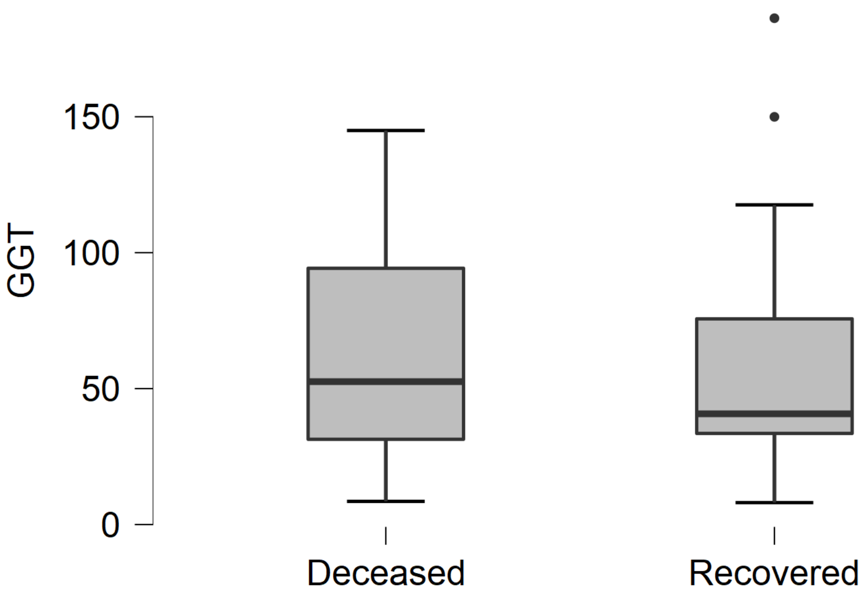
GGT
150
100
50
0
Deceased
Recovered

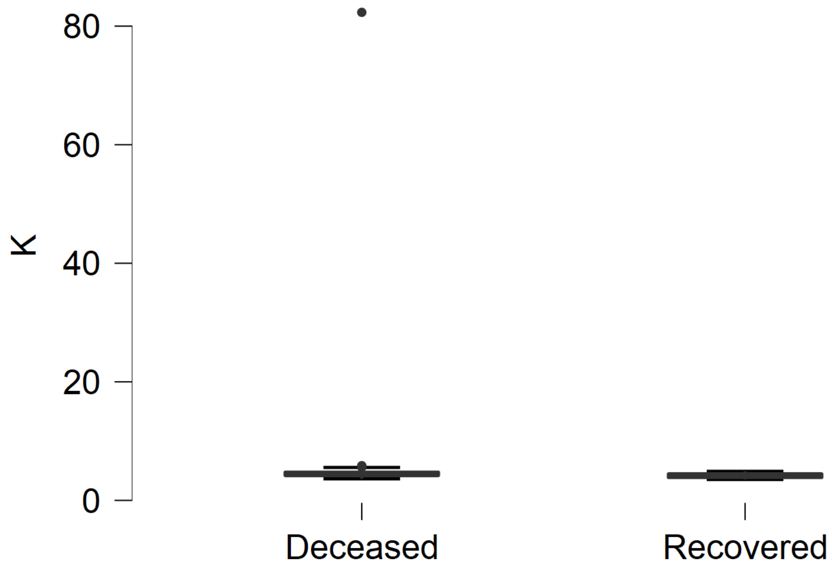
K
80
60
40
20
0
Deceased
Recovered

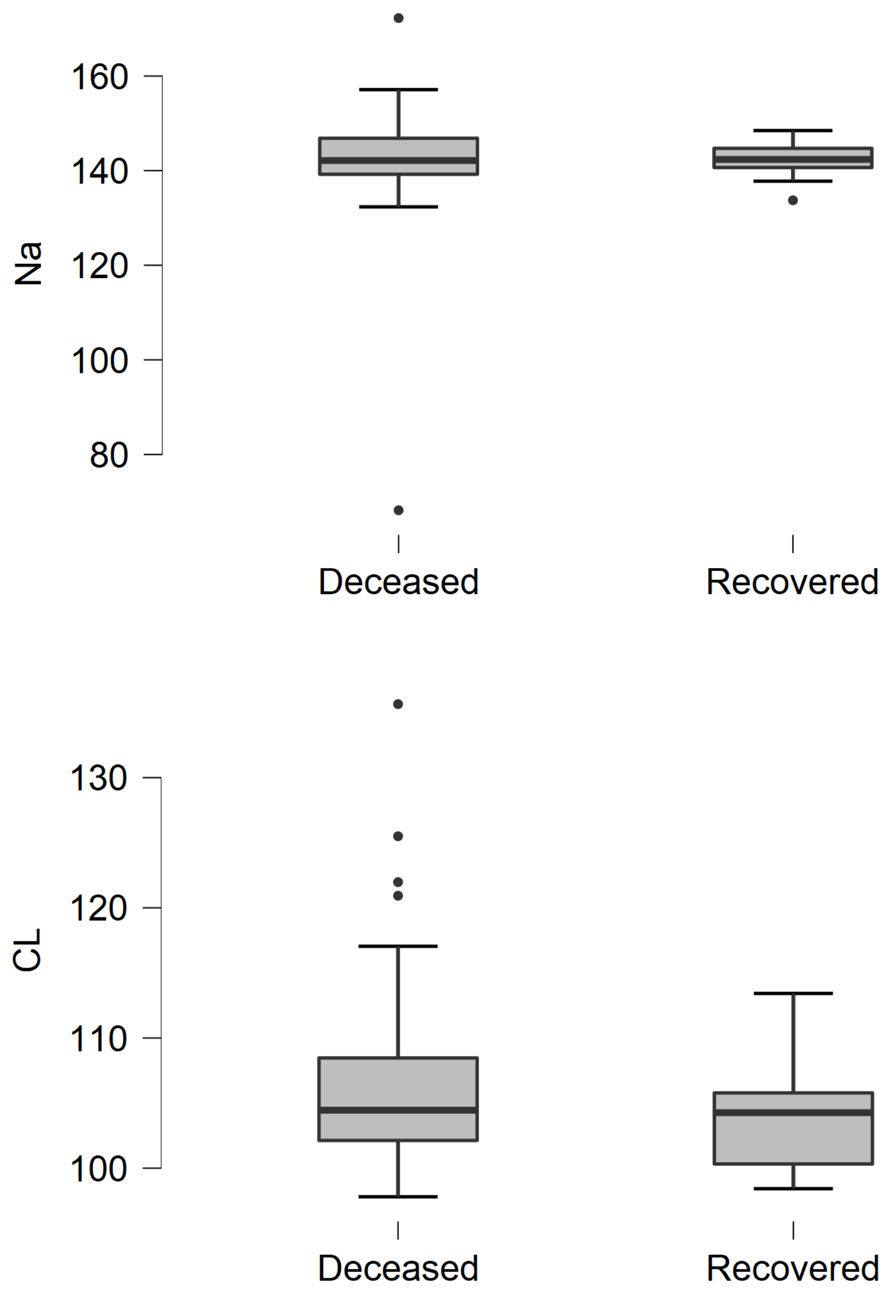
160
140
120
100
80
Na
Deceased
Recovered
130
120
110
100
CL
Deceased
Recovered

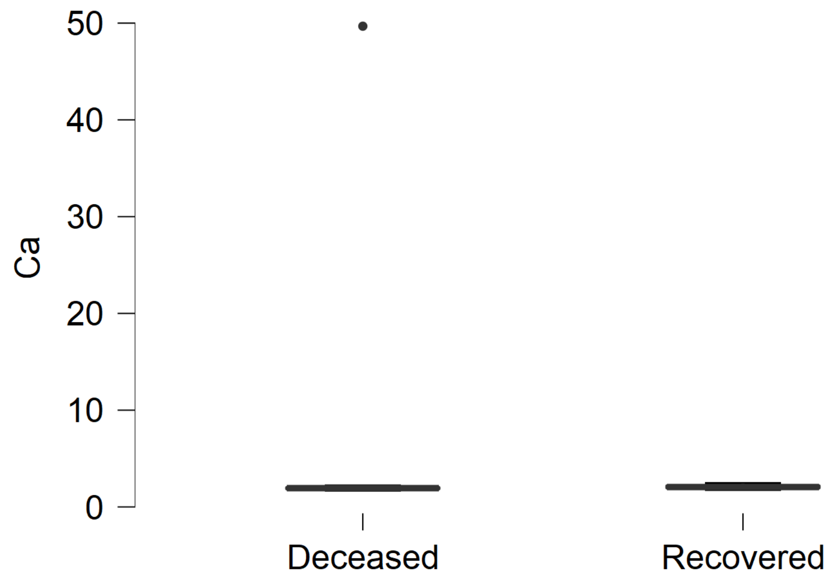
50
40
30
20
10
0
Ca
Deceased
Recovered

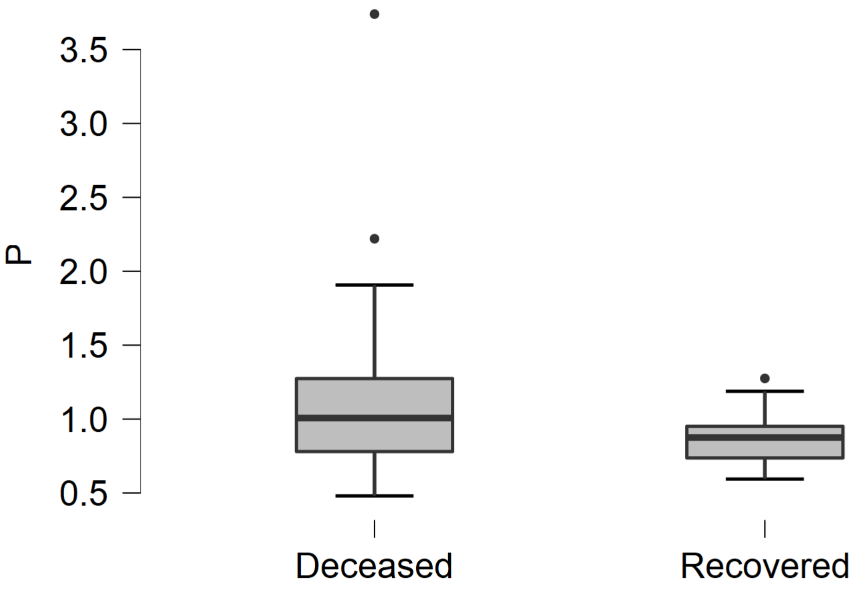
3.5
3.0
2.5
2.0
1.5
1.0
0.5
P
Deceased
Recovered

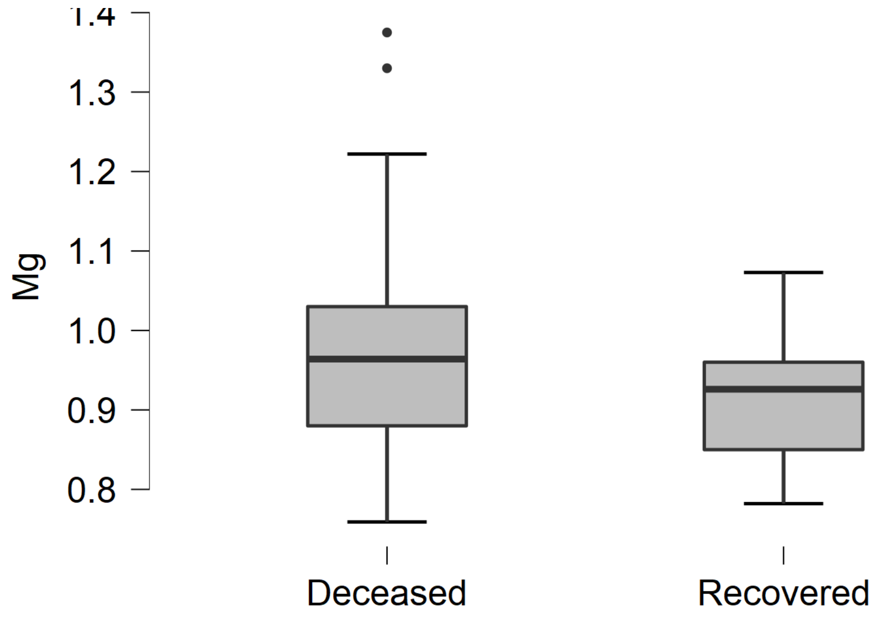

1.4
1.3
1.2
1.1
1.0
0.9
0.8
Mg
Deceased
Recovered

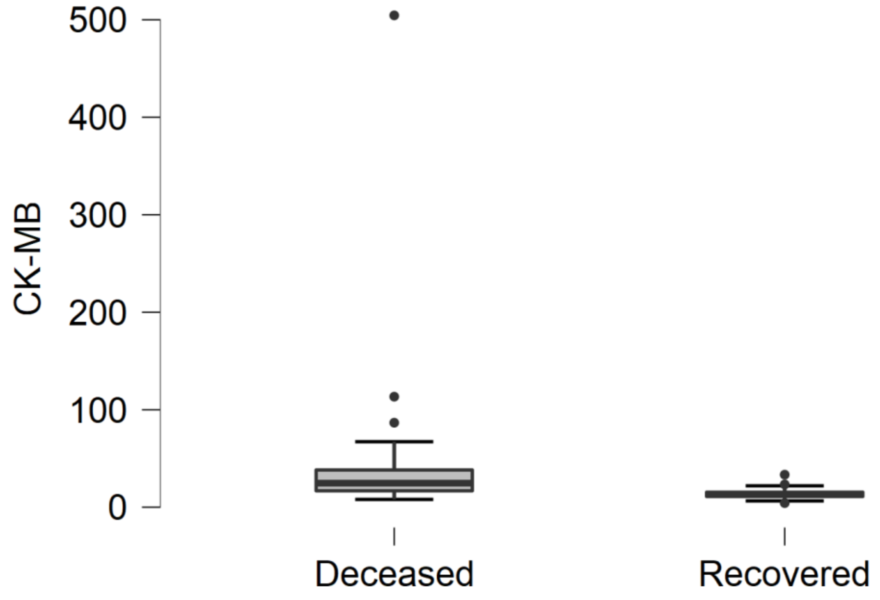

500
400
300
200
100
0
CK-MB
Deceased
Recovered

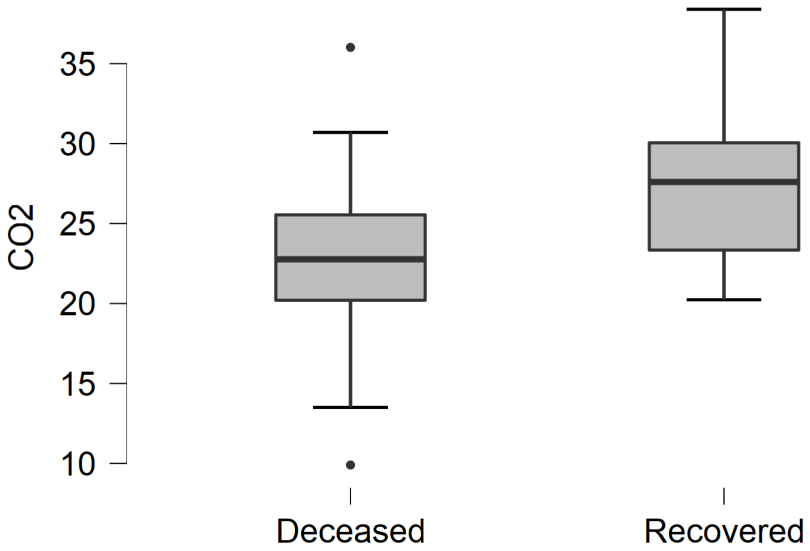
CO2
35
30
25
20
15
10
Deceased
Recovered

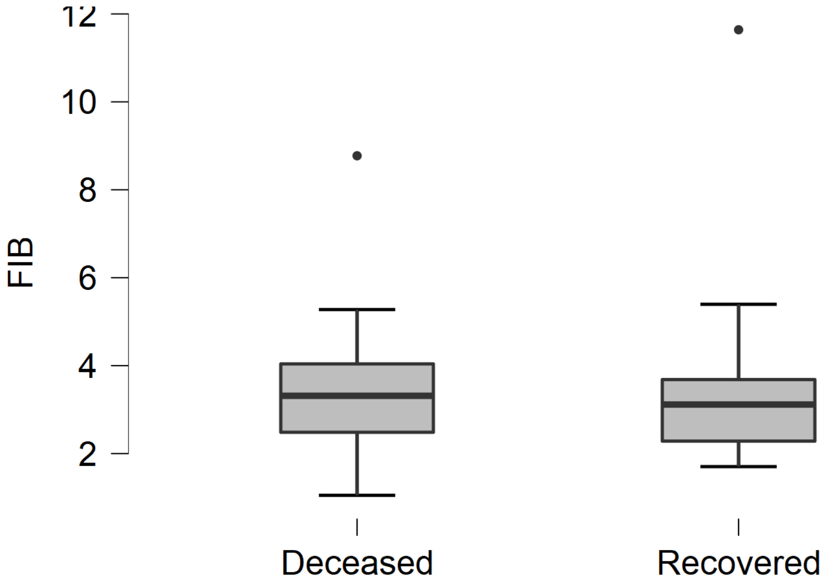
FIB
12
10
8
6
4
2
Deceased
Recovered

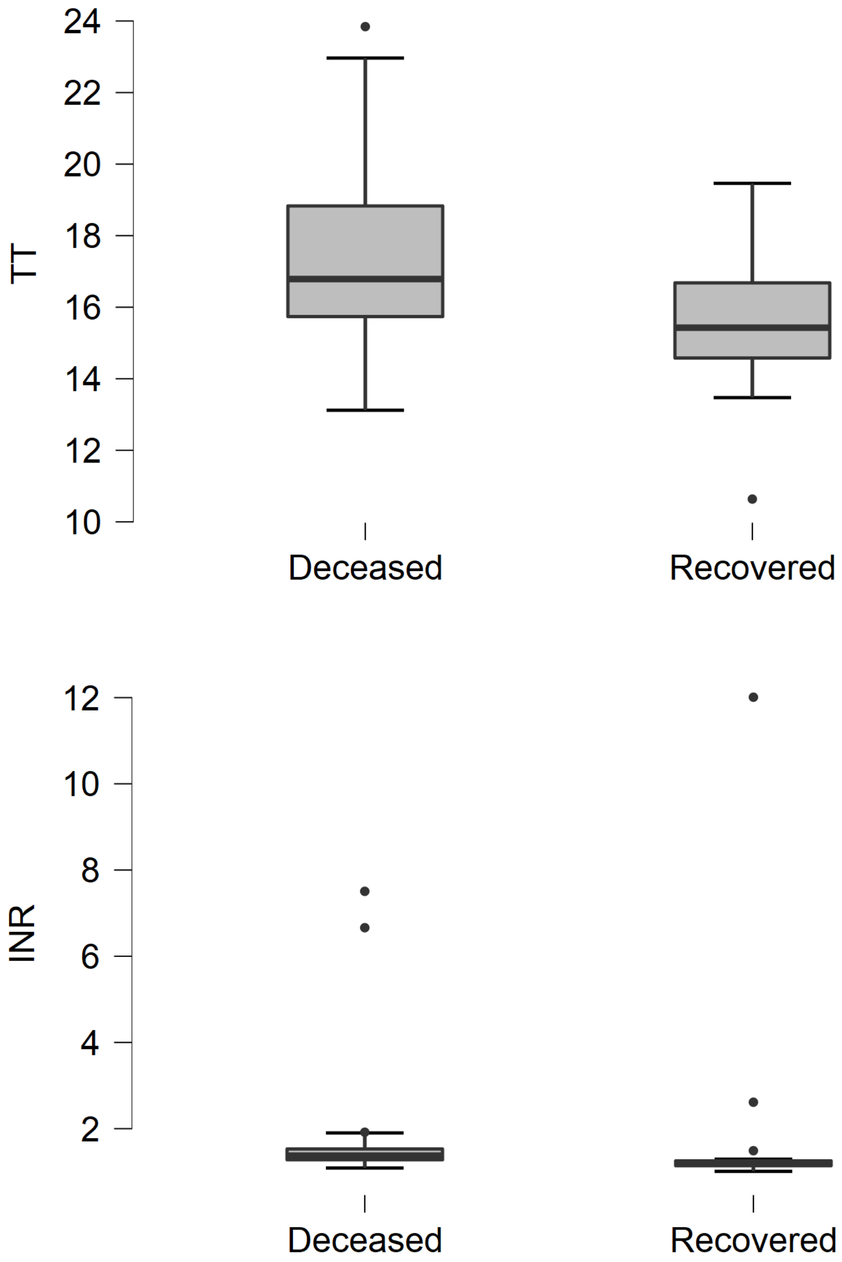

TT
24
22
20
18
16
14
12
10
Deceased
Recovered
INR
12
10
8
6
4
2
Deceased
Recovered

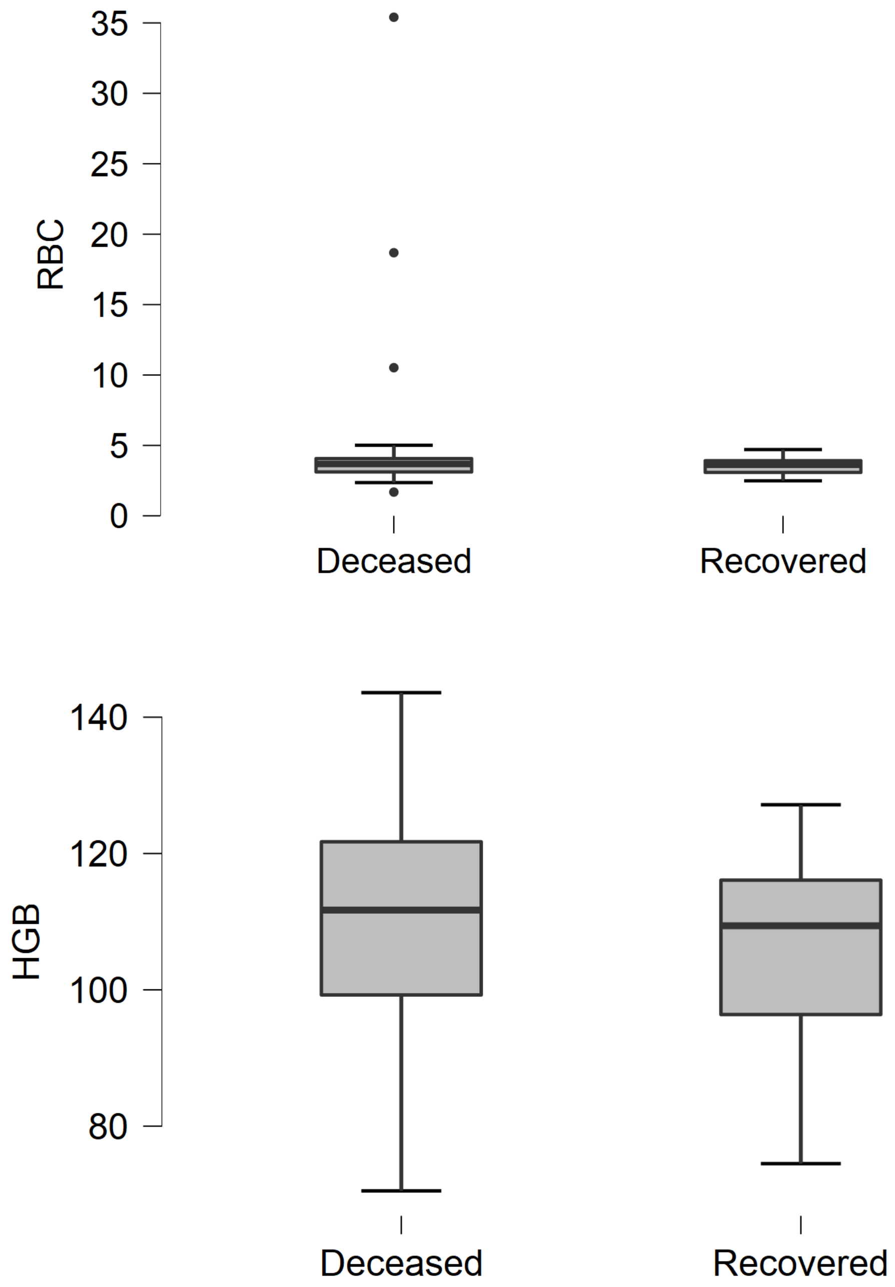
RBC
35
30
25
20
15
10
5
0
Deceased
Recovered
HGB
140
120
100
80
Deceased
Recovered

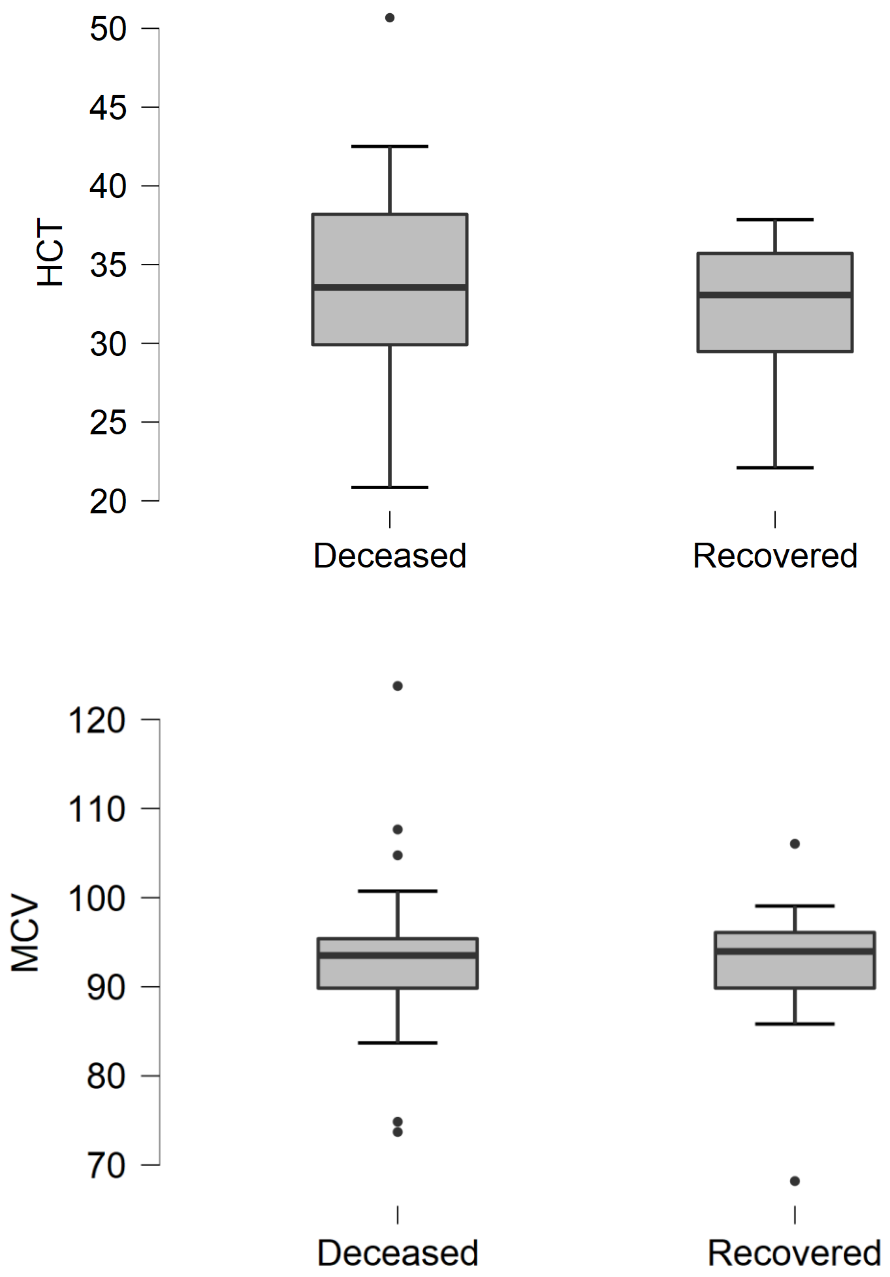

50
45
40
35
30
25
20
HCT
Deceased
Recovered
120
110
100
90
80
70
MCV
Deceased
Recovered

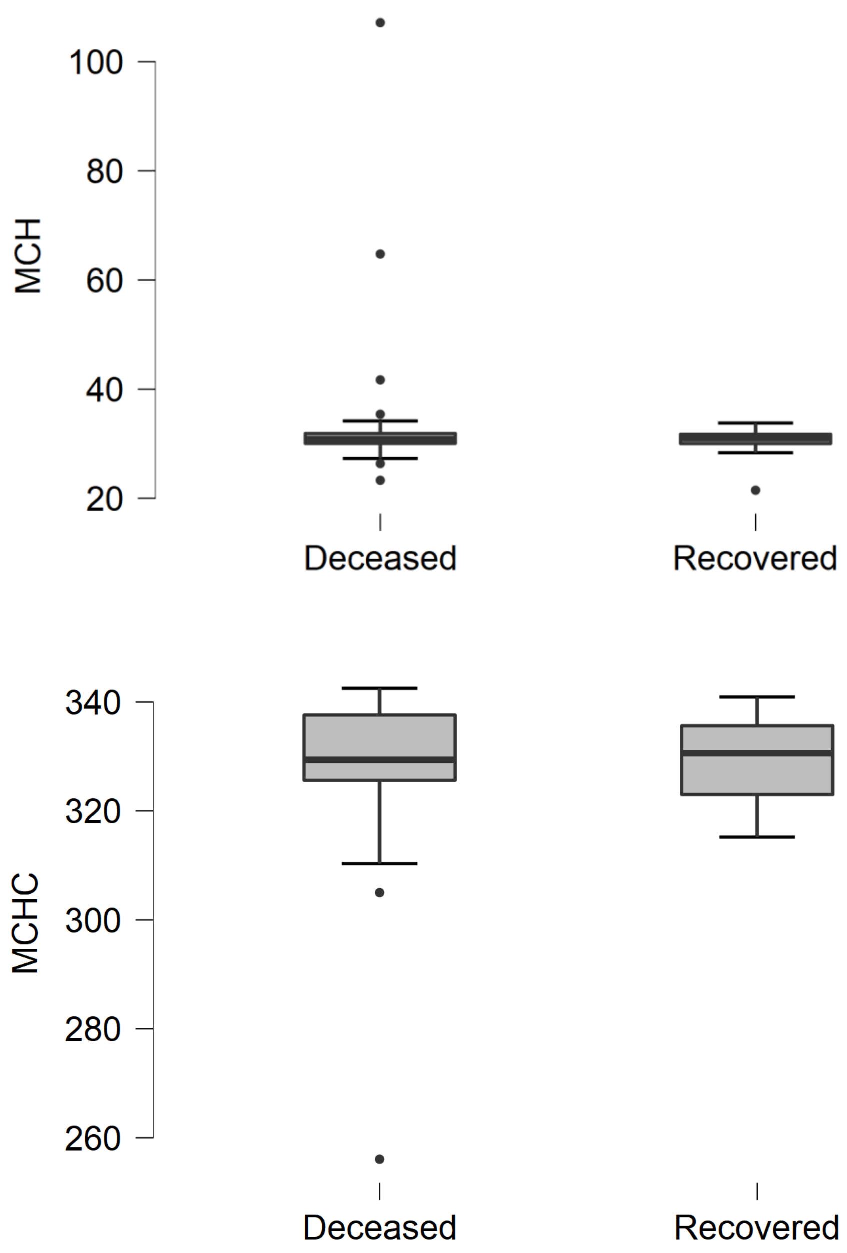

MCH
100
80
60
40
20
Deceased
Recovered
MCHC
340
320
300
280
260
Deceased
Recovered

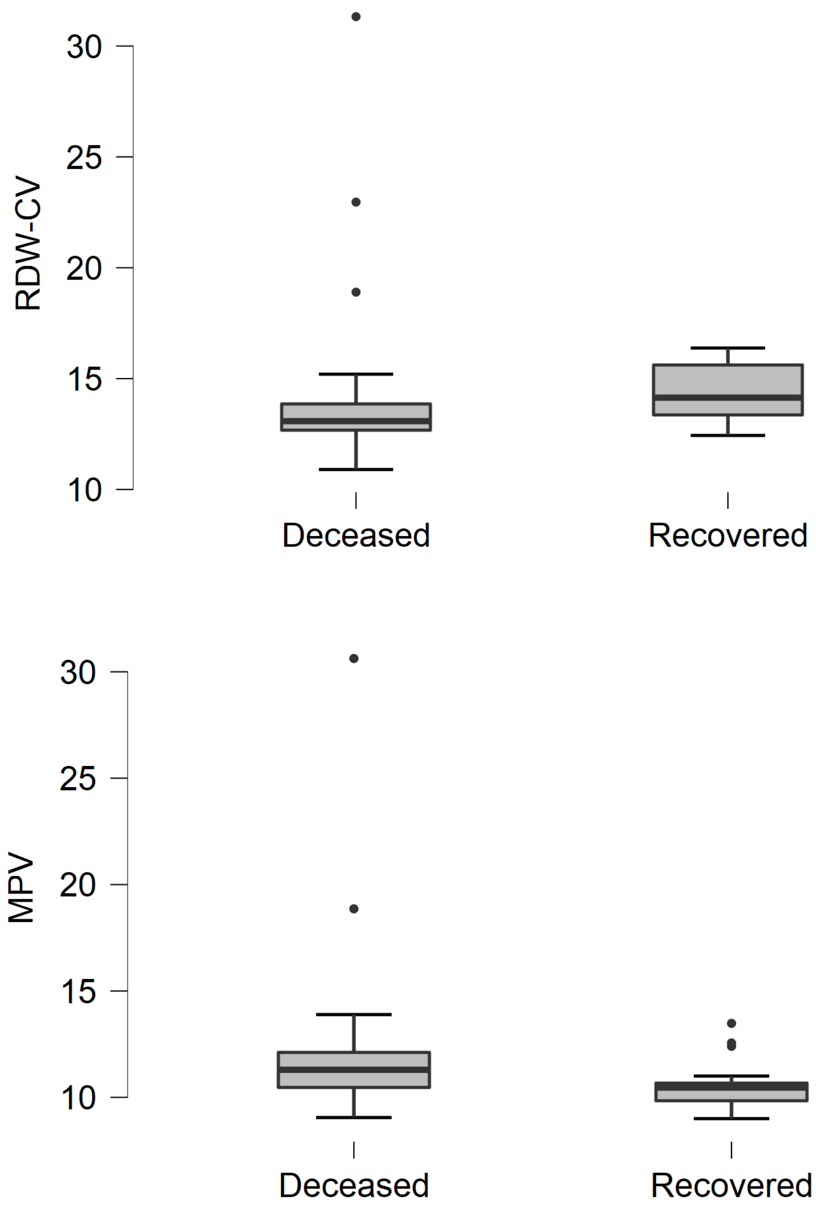
RDW-CV
30
25
20
15
10
Deceased
Recovered
MPV
30
25
20
15
10
Deceased
Recovered

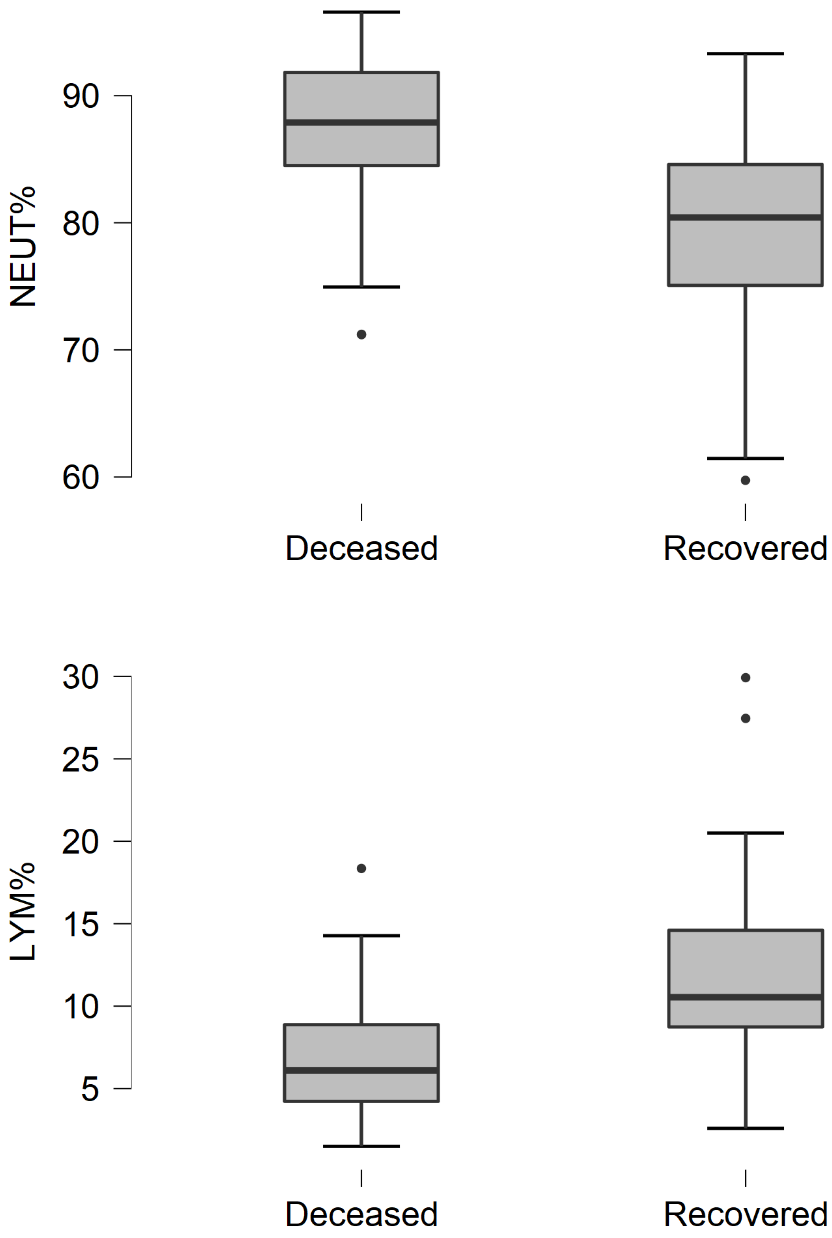

NEUT%
90
80
70
60
Deceased
Recovered
LYM%
30
25
20
15
10
5
Deceased
Recovered

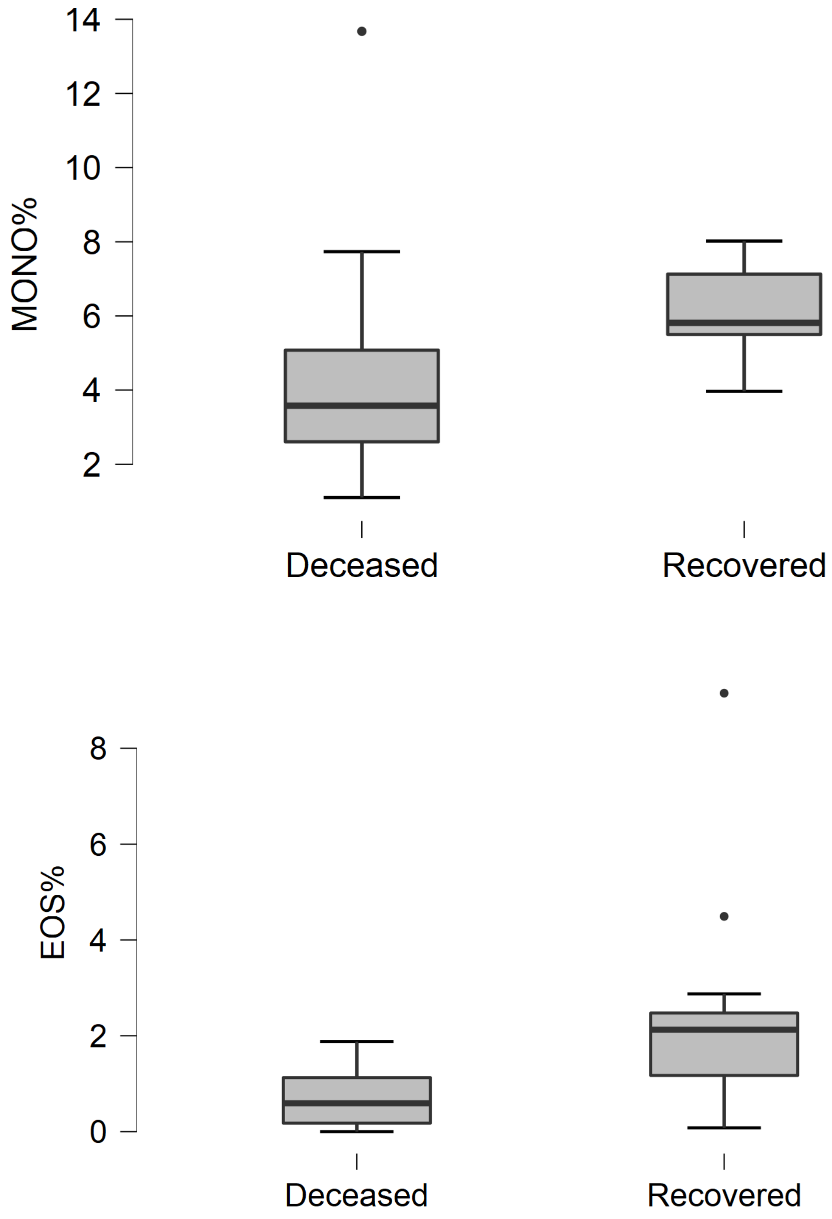
14
12
10
8
6
4
2
MONO%
Deceased
Recovered
8
6
4
2
0
EOS%
Deceased
Recovered

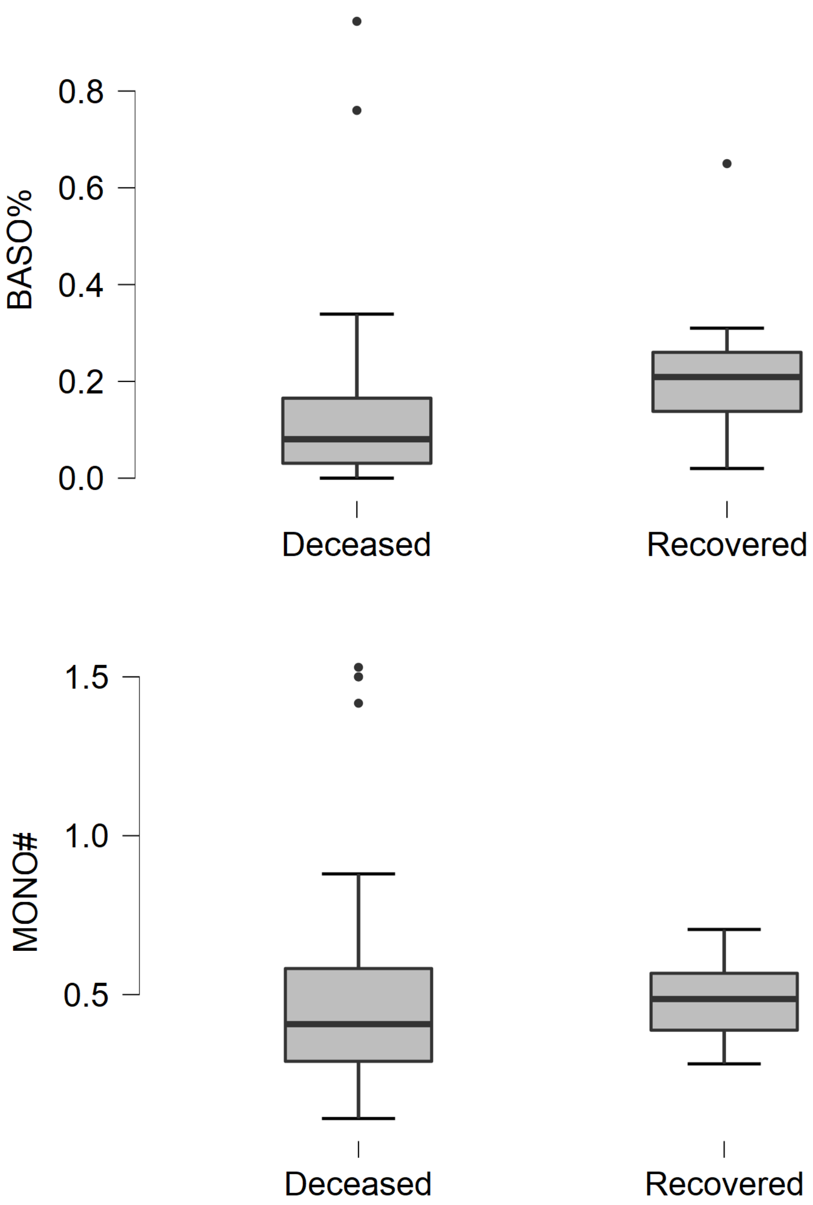
0.8
0.6
0.4
0.2
0.0
BASO%
Deceased
Recovered
1.5
1.0
0.5
MONO#
Deceased
Recovered

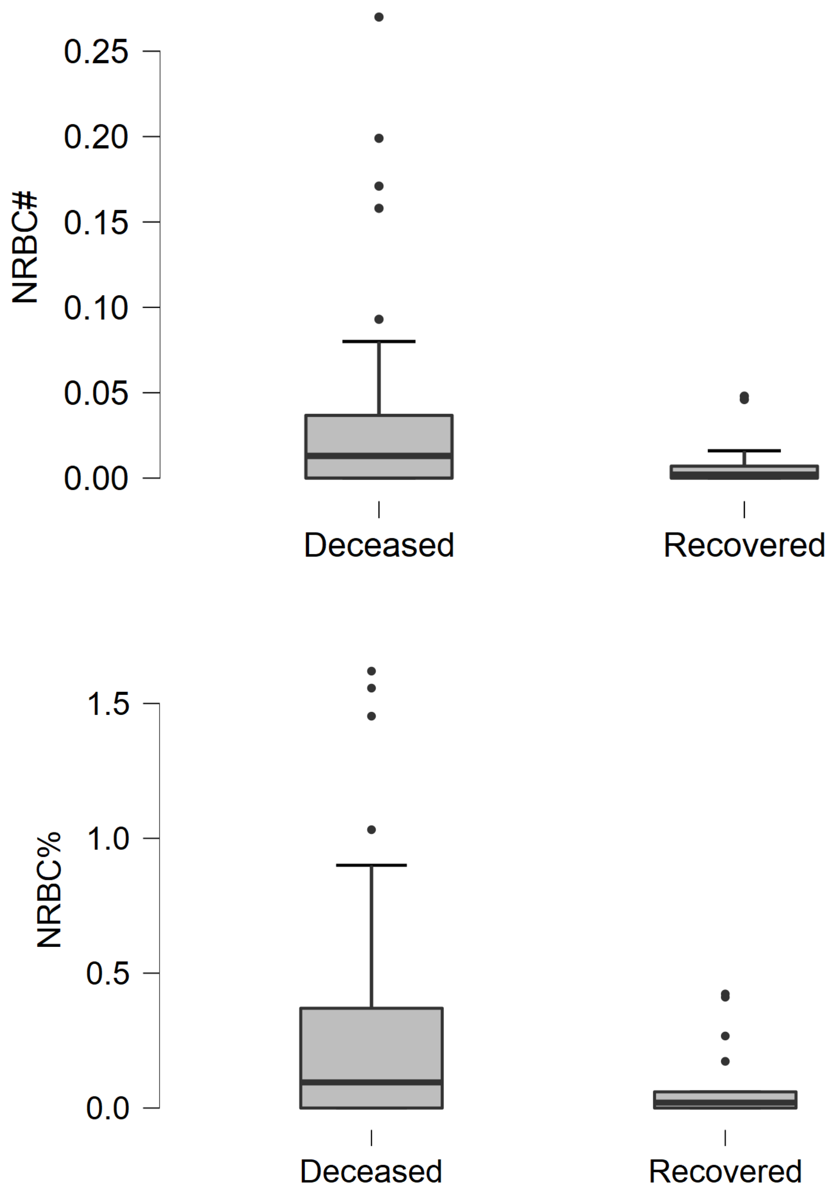
NRBC#
0.25
0.20
0.15
0.10
0.05
0.00
Deceased
Recovered
NRBC%
1.5
1.0
0.5
0.0
Deceased
Recovered

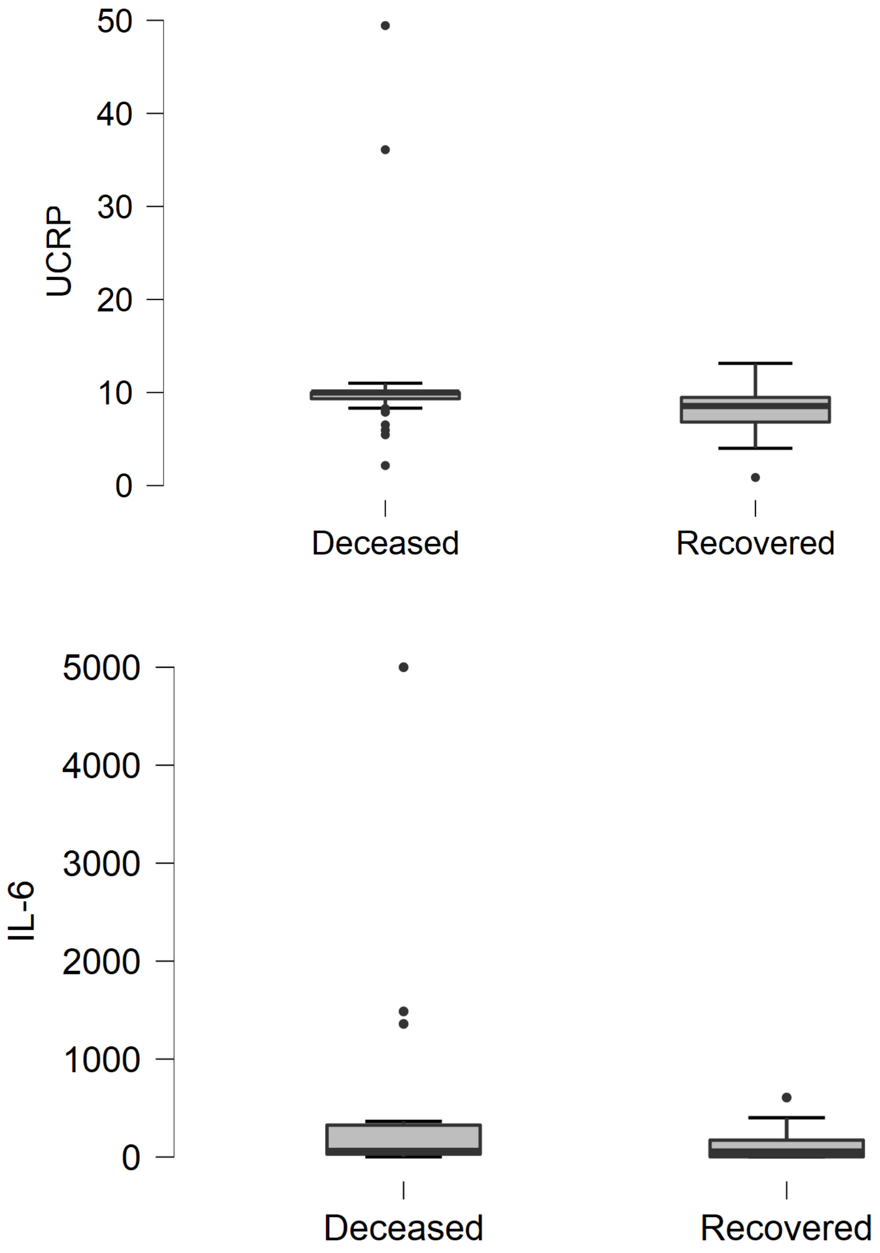

50
40
30
20
10
0
UCRP
Deceased
Recovered
5000
4000
3000
2000
1000
0
IL-6
Deceased
Recovered
